# The Zinc Finger Antiviral Protein ZAP Restricts Human Cytomegalovirus and Selectively Binds and Destabilizes Viral *UL4*/*UL5* Transcripts

**DOI:** 10.1128/mBio.02683-20

**Published:** 2021-05-04

**Authors:** Ana Cristina Gonzalez-Perez, Markus Stempel, Emanuel Wyler, Christian Urban, Antonio Piras, Thomas Hennig, Sabina Ganskih, Yuanjie Wei, Albert Heim, Markus Landthaler, Andreas Pichlmair, Lars Dölken, Mathias Munschauer, Florian Erhard, Melanie M. Brinkmann

**Affiliations:** a Viral Immune Modulation Research Group, Helmholtz Centre for Infection Research, Braunschweig, Germany; b Institute of Genetics, Technische Universität Braunschweig, Braunschweig, Germany; c Berlin Institute for Medical Systems Biology, Max-Delbrück-Center for Molecular Medicine, Berlin, Germany; d School of Medicine, Institute of Virology, Technical University of Munich, Munich, Germany; e Institute for Virology and Immunobiology, Julius-Maximilians-Universität Würzburg, Würzburg, Germany; f Helmholtz-Institute for RNA-based Infection Research, HIRI, Würzburg, Germany; g Institute of Virology, Hannover Medical School, Hannover, Germany; h IRI Life Sciences, Institute of Biology, Humboldt-Universität Berlin, Berlin, Germany; Duke University Medical Center

**Keywords:** ZAP, ZC3HAV1, ISG, antiviral, DNA virus, herpesvirus, HCMV, innate immunity, human cytomegalovirus, interferons, mRNA degradation, pattern recognition receptors, zinc finger proteins

## Abstract

Interferon-stimulated gene products (ISGs) play a crucial role in early infection control. The ISG zinc finger CCCH-type antiviral protein 1 (ZAP/ZC3HAV1) antagonizes several RNA viruses by binding to CG-rich RNA sequences, whereas its effect on DNA viruses is less well understood. Here, we decipher the role of ZAP in the context of human cytomegalovirus (HCMV) infection, a β-herpesvirus that is associated with high morbidity in immunosuppressed individuals and newborns. We show that expression of the two major isoforms of ZAP, ZAP-S and ZAP-L, is induced during HCMV infection and that both negatively affect HCMV replication. Transcriptome and proteome analyses demonstrated that the expression of ZAP results in reduced viral mRNA and protein levels and decelerates the progression of HCMV infection. Metabolic RNA labeling combined with high-throughput sequencing (SLAM-seq) revealed that most of the gene expression changes late in infection result from the general attenuation of HCMV. Furthermore, at early stages of infection, ZAP restricts HCMV by destabilizing a distinct subset of viral mRNAs, particularly those from the previously uncharacterized *UL4-UL6* HCMV gene locus. Through enhanced cross-linking immunoprecipitation and sequencing analysis (eCLIP-seq), we identified the transcripts expressed from this HCMV locus as the direct targets of ZAP. Moreover, our data show that ZAP preferentially recognizes not only CG, but also other cytosine-rich sequences, thereby expanding its target specificity. In summary, this report is the first to reveal direct targets of ZAP during HCMV infection, which strongly indicates that transcripts from the *UL4-UL6* locus may play an important role for HCMV replication.

## INTRODUCTION

Viral infections pose a major global health burden as the cause of a range of debilitating human diseases with the potential to paralyze countries. Herpesviruses are large, structurally complex DNA viruses belonging to the *Herpesviridae*. Within this family, a number of viruses are responsible for a variety of diseases in humans ranging from cold sores and pneumonia to cancer. The common peculiarity of herpesviruses lies in their ability to establish latency, which presents a particular challenge due to severe complications resulting from virus reactivation. Human cytomegalovirus (HCMV), part of the *Betaherpesvirinae* subfamily, is one of the nine human herpesviruses described to date. HCMV displays a coding capacity that exceeds that of most other *Herpesviridae*, having the largest genome among all known human viruses and the capacity to encode more than 200 proteins. Primary HCMV infection generally causes mild symptoms in immunocompetent individuals (1). However, immunosuppressed individuals, such as AIDS patients or transplant recipients, are vulnerable to HCMV-related disease (2, 3). In addition, HCMV is the leading cause of congenital viral infection worldwide and can result in serious long-term sequelae in newborns, such as hearing loss, vision abnormalities, microcephaly, or developmental delays (4).

The host innate immune system, as the first line of defense, is equipped with germ line-encoded pattern recognition receptors (PRRs), a group of sensors that detect the presence of pathogens by recognizing pathogen-associated molecular patterns (PAMPs). The detection of PAMPs induces downstream signaling culminating in the activation of several transcription factors, including interferon regulatory factors (IRF) and nuclear factor kappa-light-chain-enhancer of activated B cells (NF-κB), leading to the induction of genes encoding type I interferons (IFNs), proinflammatory cytokines, and noncanonical interferon-stimulated genes (ISGs) (5). Upon binding type I IFNs, the interferon-α/β receptor (IFNAR) is activated, and its signaling results in nuclear translocation of STAT1 and STAT2 transcription factors and induction of canonical ISGs (reviewed in reference 6). ISGs are essential antiviral effectors and constitute a group of cellular factors ranging from PRR (e.g., IFI16, cGAS, or RIG-I) or transcription factors to proapoptotic proteins or proteins involved in the regulation of the immune response (7).

The zinc finger CCCH-type antiviral protein 1, also known as ZAP, ZC3HAV1, or PARP13, belongs to the subset of noncanonical ISGs whose expression can be induced via IRF3 directly as well as canonically by IFNAR signaling (5). Four isoforms of ZAP that originate from alternative splicing of the *ZC3HAV1* gene have been reported thus far (8), with the long (ZAP-L) and the short (ZAP-S) isoforms being the most prominent ones. While approximately the first 700 amino acids are shared by ZAP-L and ZAP-S, ZAP-L has an extended C terminus of around 200 amino acids containing a catalytically inactive PARP-like domain (9) and a functional CaaX prenylation motif (10). The farnesyl modification on the cysteine residues of the CaaX motif increases the hydrophobicity of ZAP-L, targeting this isoform to endolysosomal membranes (10). Both ZAP isoforms are equipped with an N-terminal zinc finger domain (containing four CCCH-type zinc finger motifs), a TiPARP homology domain (TPH), which is well conserved among ZAP paralogs and contains a fifth zinc finger motif, and a WWE domain, predicted to mediate specific protein-protein interactions (11, 12).

ZAP exhibits broad antiviral activity against a variety of RNA viruses by binding RNA and mediating its degradation (13–16). The antiviral activity of ZAP was demonstrated against alphaviruses (17), filoviruses (18), retroviruses (16, 19), and flaviviruses (20). However, ZAP fails to inhibit a diverse range of other RNA viruses, including vesicular stomatitis virus (VSV), poliovirus (17), influenza A virus (21, 22), and enterovirus A71 (23). The involvement of ZAP in the defense against DNA viruses has not been explored to the same extent as for RNA viruses. For instance, ZAP restricts hepatitis B virus by downregulating viral pregenomic RNA (24). For herpesviruses, while ectopic expression of ZAP failed to inhibit growth of the α-herpesvirus herpes simplex virus 1 (HSV-1) (17), ZAP could restrict HCMV by an unknown mechanism (25). Interestingly, a luciferase-based reporter assay identified the HSV-1 UL41 protein, known for its ability to mediate degradation of several mRNAs, as a ZAP antagonist that degrades *ZAP* mRNA, which may explain why ZAP cannot restrict HSV-1 (17, 26). Modified vaccinia virus Ankara (MVA) was recently shown to be restricted by ZAP, and while the knockout of ZAP had no discernible effect on viral DNA, individual mRNA, or protein species, an interference of ZAP with a late step in the assembly of infectious MVA virions was suggested (27).

To date, the molecular details of how ZAP recognizes RNA have not been clearly defined. Early publications suggest an RNA structure-dependent recognition, based on the RNA tertiary structure, but already advocating the importance of the sequence-specific interaction between ZAP and its target RNA (28). The formation of tertiary structures raises the possibility of multiple binding sites. Subsequent studies support the recognition of CG-rich dinucleotide regions (19). Indeed, a recent study revealed on a structural level that ZAP binds to CG-rich RNA with high affinity through its basic second zinc finger, which contains a pocket capable of accommodating CG-dinucleotide bases (29). However, another possibility is the binding of ZAP to AU-dinucleotides. Although one study reported that ZAP does not recognize any of the three types of AU-rich elements (AREs) and concluded that ZAP may modulate stability of non-ARE-containing mRNAs (14), recent publications described ZAP target specificity for sequences enriched for AU-rich dinucleotides (30, 31). Taken together, while there are solid data indicating ZAP recognition of CG-dinucleotide-containing RNA, it is feasible that the presence of several other zinc finger motifs in the ZAP protein broadens its target specificity. How these target specificities are regulated and the mechanism of how ZAP-bound RNA is degraded warrant further investigation.

Here, we report that the expression of the ZAP-S and ZAP-L isoforms is induced upon infection and that both act as restriction factors for HCMV in human primary fibroblasts (HFF-1). Further, a combination of transcriptomic and proteomic experiments demonstrates that ZAP restricts the progression of HCMV gene expression, consequently affecting viral protein levels. Enhanced cross-linking and immunoprecipitation (eCLIP) revealed that ZAP preferentially cross-links to cytosine-rich regions devoid of adenosines, rather than recognizing specific motifs. In combination with metabolic RNA labeling (SLAM-sequencing) we show that ZAP negatively regulates the stability of the viral transcripts *UL4* and *UL5* by direct interaction. Together, we provide evidence that ZAP is an important cellular factor that restricts HCMV early during infection in a distinct manner, by specifically targeting transcripts that originate from the *UL4-UL6* locus expressed with immediate-early to early kinetics.

## RESULTS

### Expression of ZAP is induced upon HCMV infection.

**FIG 1:**
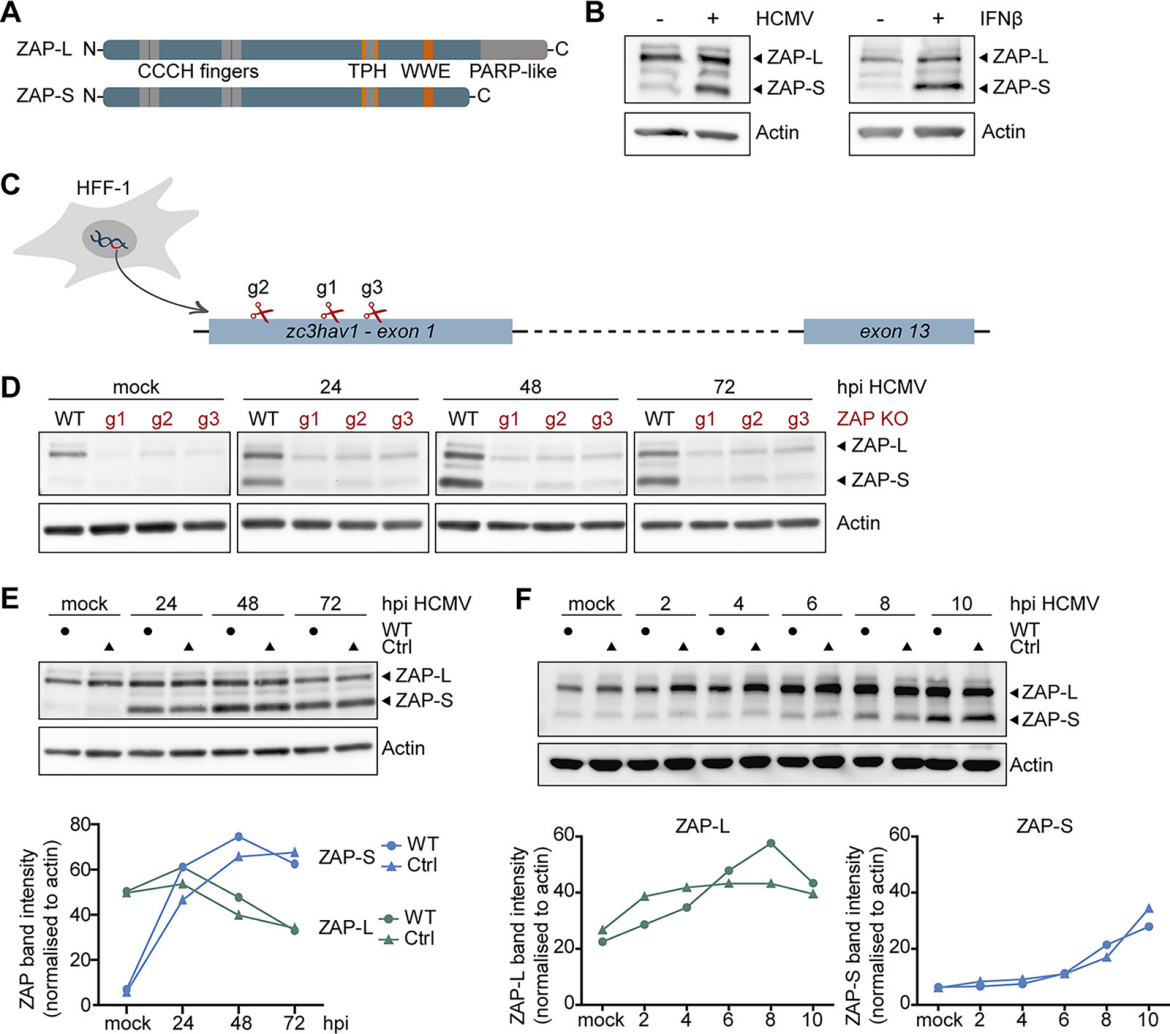
Expression kinetics of ZAP-S and ZAP-L in HCMV-infected fibroblasts. (A) Schematic representation of the protein domains of the two main isoforms of ZAP, the long isoform, ZAP-L, and the short isoform, ZAP-S. Both isoforms share four CCCH-type zinc finger motifs in the N-terminal domain, as well as a TPH domain containing a fifth zinc finger motif, and a WWE domain, while the C-terminal PARP-like domain is only present in ZAP-L. TPH, TiPARP homology domain; PARP, poly(ADP-ribose)-polymerase. (B) Primary human fibroblasts (HFF-1) were either mock-treated, infected by centrifugal enhancement with HCMV (MOI 0.1), or stimulated by addition of recombinant IFN-β (20 ng/ml), and expression of ZAP and actin was analyzed 24 h later by immunoblotting with a ZAP- or actin-specific antibody. (C) Three independent ZAP KO cell lines were generated by Cas9-mediated gene editing using three different gRNAs (g1, g2, and g3) that target the first exon of the *zc3hav1* gene. (D to F) Wild-type HFF-1 and the three ZAP KO (D) or control (E and F) cell lines were mock-treated or infected by centrifugal enhancement with HCMV (MOI 0.1) for the indicated time points, and cell lysates were subjected to immunoblotting with specific antibodies against ZAP and actin. Quantifications of ZAP-L (in green) or ZAP-S (in blue) band intensities normalized to actin are represented in line graphs. hpi, hours postinfection.

A previous proteomics study showed that HCMV infection of human fibroblasts (HFF-1) leads to the upregulation of a specific set of host proteins in the first 24 h, including some ISGs. One of these ISGs was the antiviral protein ZAP (32). The two major isoforms of ZAP, the short (ZAP-S) and the long (ZAP-L), are only differentiated by the inclusion of the C-terminal PARP-like domain in ZAP-L (Fig. 1A). To analyze which of the two major isoforms of ZAP is induced upon HCMV infection, HFF-1 cells were infected with HCMV or, as a control, stimulated with recombinant IFN-β, and expression of ZAP was analyzed by immunoblotting. Our results show that ZAP-L was expressed in uninfected HFF-1 cells, and its expression was only marginally increased upon HCMV infection or IFN-β 24 h poststimulation (Fig. 1B). In contrast, ZAP-S was barely expressed in untreated cells, but its expression was strongly induced by HCMV infection and IFN-β treatment (Fig. 1B). These results show that ZAP-S is a prototypical ISG, which is strongly induced upon HCMV infection, whereas ZAP-L is already expressed prior to, and only slightly induced with, infection.

To investigate whether ZAP can shape the course of HCMV infection, we generated three individual ZAP-deficient HFF-1 cell lines by Cas9-mediated gene editing. Each cell line was generated using a different guide RNA, all targeting the first exon of *Zc3hav1*, and thereby affecting both ZAP-L and ZAP-S expression (Fig. 1C). To verify the efficacy of genome editing, we infected the knockout (KO) cell lines with HCMV and analyzed ZAP expression at 24, 48, and 72 h postinfection (hpi). Protein levels of both ZAP-L and ZAP-S were strongly reduced in all three knockout cell lines (g1, g2, g3), confirming successful genome editing (Fig. 1D). As a control, we also generated a cell line with a nontargeting guide RNA. Expression of ZAP followed the same kinetics in both wild-type (WT) and control HFF-1 cells, demonstrating that stable expression of Cas9 did not affect ZAP expression kinetics (Fig. 1E). To pinpoint when ZAP-S expression begins to increase after HCMV infection, we monitored its protein levels in WT and control cells at earlier time points (2 to 10 hpi). ZAP-S expression levels started to be detectable between 6 and 8 h post-HCMV infection and steadily increased over time (Fig. 1F).

Taken together, these results show that HCMV infection leads to a steady increase of ZAP-S levels, while ZAP-L is already expressed in uninfected cells but also further induced upon infection and is overall stable throughout a complete cycle of HCMV replication.

### Both ZAP-S and ZAP-L negatively affect HCMV genome replication.

**FIG 2:**
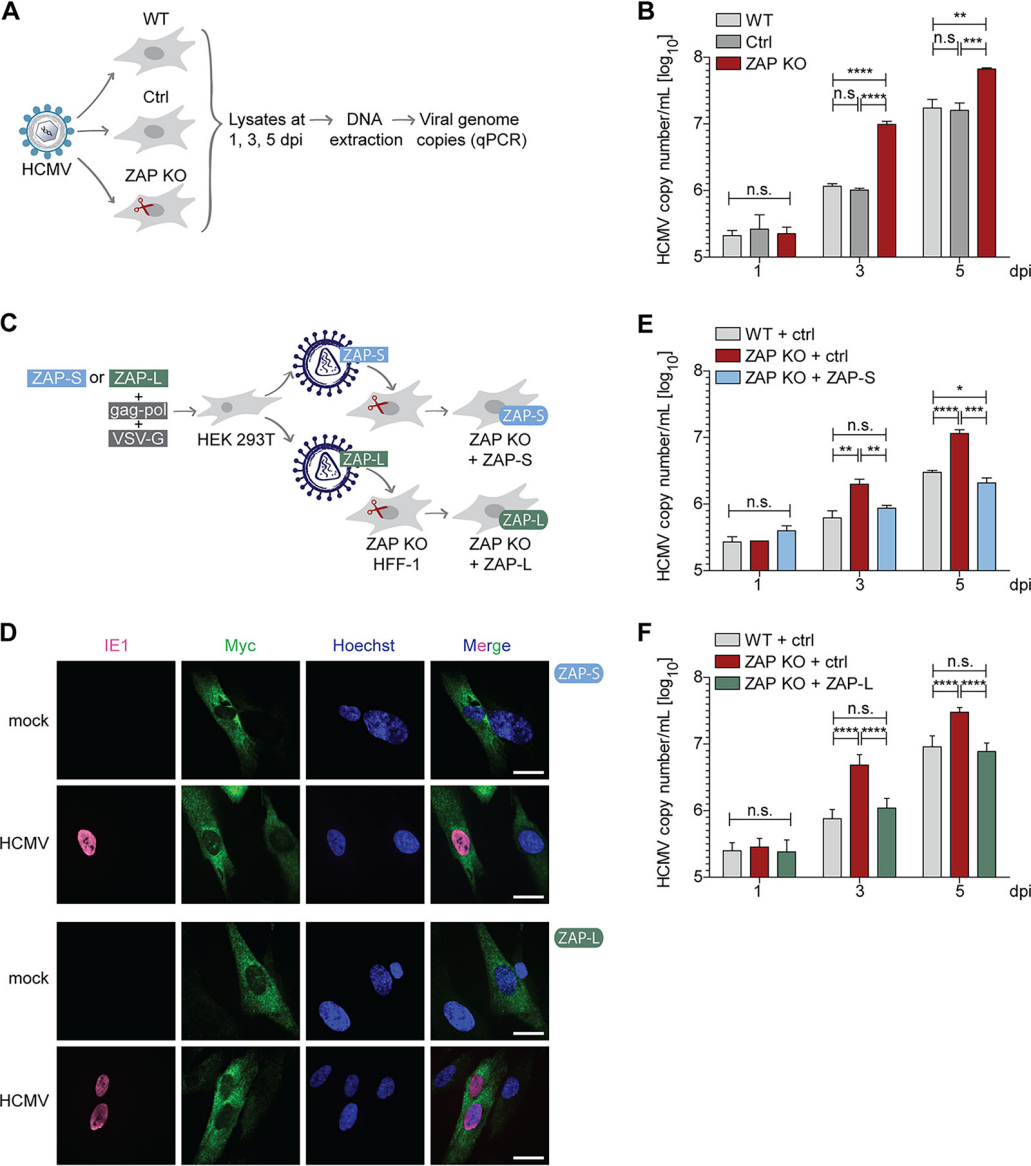
ZAP-S and ZAP-L restrict HCMV replication in HFF-1 cells. (A) Schematic representation of the workflow to determine HCMV genome copy numbers. WT, control, or ZAP KO HFF-1 cells were infected with HCMV (MOI 0.1) for 2 h. Both cells and supernatant were harvested at 1, 3, and 5 days postinfection (dpi), followed by DNA extraction and measurement of viral genome copies by quantitative PCR. (B) HCMV genome copy numbers from WT, control, or ZAP KO HFF-1 cells were determined as described in panel A. HCMV copy numbers/ml are displayed as bar plots showing the mean ± standard deviation (S.D.) of biological triplicates. Results shown are one representative of at least three independent experiments using two different ZAP KO cell lines, with similar results obtained in all replicates. (C) Schematic representation of the workflow to reconstitute ZAP KO HFF-1 cells. HEK 293T cells were transfected with either myc-tagged ZAP-S or ZAP-L expression plasmids together with the packaging (gag-pol) and the envelope (VSV-G) plasmids to produce lentiviruses harboring ZAP-S or ZAP-L, respectively, followed by transduction of ZAP KO HFF-1 cells. As the control, WT and ZAP KO HFF-1 cells were transduced with lentiviruses harboring empty vector. (D) Subcellular localization of myc-tagged ZAP-S and ZAP-L in ZAP KO HFF-1 cells. ZAP KO cells were transduced as described in panel C with either myc-tagged ZAP-S or ZAP-L. Transduced cells were infected by centrifugal enhancement with HCMV (MOI 0.1), and 24 h postinfection, cells were fixed for immunolabeling with myc- and HCMV IE1-specific antibodies. IE1, immediate-early protein 1. Nuclei were stained with Hoechst 33342. Scale bars represent 50 μm. (E and F) HCMV genome copy numbers from WT, ZAP KO, or ZAP KO HFF-1 cells reconstituted with ZAP-S (E) or ZAP-L (F) were determined as described in panel A. HCMV copy numbers/ml are displayed as bar plots showing the mean ± S.D. of one (E) or two combined independent (F) experiments performed with biological triplicates. Two independent experiments for both ZAP-S and ZAP-L were performed. Significant changes were calculated using unpaired two-sided Student’s *t* tests, n.s., not significant; *, *P < *0.05; **, *P < *0.01; ***, *P < *0.001; ****, *P < *0.0001.

To investigate the impact of ZAP on HCMV replication, WT, control, or ZAP KO HFF-1 cells were infected with HCMV, and viral genome copies were quantified by quantitative PCR at 1, 3, and 5 days postinfection (dpi) (Fig. 2A). We included day 1 of infection in our analysis to verify that the KO of ZAP is not affecting viral entry and that the different cell lines were infected to a similar extent. Indeed, at this early time point when HCMV has not entered the first round of viral genome replication, no significant differences in the number of HCMV genome copies were detected (Fig. 2B). At day 3 postinfection, when HCMV has completed its first replication cycle, WT and control cells showed significantly lower numbers of viral genome copies compared to ZAP KO cells. At 5 days postinfection, HCMV genome copy numbers in WT and control cells were still 5 times lower than those in ZAP KO cells (Fig. 2B). These results suggest that HCMV genome replication is negatively affected by ZAP-S, ZAP-L, or both.

Next, we sought to elucidate which of the two major isoforms contributes to the restriction of HCMV replication. To address this, we reconstituted ZAP KO cells by lentiviral transduction with either an empty vector control or C-terminally myc-tagged forms of ZAP-S or ZAP-L (Fig. 2C). Both ZAP isoforms were codon optimized to avoid recognition and cleavage by the stably expressed guide RNA (gRNA) and Cas9 within the KO cell lines. Protein levels of both codon-optimized and WT isoforms were comparable when expressed in HEK 293T cells, showing that codon optimization of the ZAP-S or ZAP-L gene did not negatively affect protein expression (Fig. S1). Upon stable expression in ZAP KO HFF-1 cells, both ZAP-S and ZAP-L localized to the cytoplasm under both uninfected and infected conditions (Fig. 2D). Next, the reconstituted ZAP KO cells were infected with HCMV, and genome copy numbers were analyzed as described above (Fig. 2A). Strikingly, reconstitution with either ZAP-S (Fig. 2E) or ZAP-L (Fig. 2F) in ZAP KO cells rescued the phenotype. While HCMV genome copy numbers were equal in infected WT and ZAP-S or ZAP-L reconstituted cells, ZAP KO cells showed significantly higher viral copy numbers. To exclude the possibility that the C-terminal myc-tag of ZAP-L could be interfering with the CaaX prenylation motif and therefore with prenylation of ZAP-L and its correct subcellular localization, we analyzed the viral genome copy numbers in ZAP KO cells expressing an untagged version of ZAP-L. As shown in Fig. S2A, myc-tagged and untagged ZAP-L had similar effects on HCMV replication.

10.1128/mBio.02683-20.5 FIG S1 Codon-optimization of ZAP does not affect ZAP protein levels. HEK 293T cells were transfected with either pEF empty vector (ev), pEF1-ZAP-S-myc (WT), or pEF1-ZAP-S-myc codon-optimized (opt.) (upper panel) or with pEF1-ZAP-L-myc WT or codon-optimized (opt.) expression constructs (lower panel). Expression levels of ZAP were determined by immunoblotting using a ZAP-specific antibody. Actin served as the loading control. Download FIG S1, TIF file, 0.1 MB. Copyright © 2021 Gonzalez-Perez et al. 2021 Gonzalez-Perez et al. https://creativecommons.org/licenses/by/4.0/ This content is distributed under the terms of the Creative Commons Attribution 4.0 International license.

10.1128/mBio.02683-20.6 FIG S2 The untagged version of ZAP-L can control HCMV replication. (A) Schematic representation of the protein domains of the two main isoforms of ZAP (long ZAP-L and short ZAP-S) as described in Fig. 1A. The CaaX prenylation motif, only present in ZAP-L, is indicated with a green arrow. C, cysteine; aa, aliphatic amino acids; X, one of several amino acids. (B, C) WT, ZAP KO, or ZAP KO HFF-1 cells reconstituted with myc-tagged ZAP-S or untagged ZAP-L were generated as described in Fig. 2C. As the control, WT and ZAP KO HFF-1 cells were transduced with lentiviruses harboring empty vector (ctrl). (B) HCMV genome copy numbers were determined as shown in Fig. 2A. Briefly, cells were infected with HCMV (MOI 0.1) for 2 h. Both cells and supernatant were harvested at 1, 3, and 5 days postinfection (dpi), followed by DNA extraction and measurement of viral genome copies by qPCR. HCMV copy numbers/ml are displayed as bar plots showing the mean ± S.D. of one independent experiment performed with biological triplicates. (C) Cells were infected by centrifugal enhancement with HCMV (MOI 0.1), and lysates were analyzed at the indicated time points postinfection by immunoblotting with specific antibodies against ZAP, HCMV UL44, and actin. Quantifications of UL44 band intensities normalized to actin are indicated and represented as bar plots. WT (in gray) or ZAP KO (in red) transduced with ctrl, empty vector; S, ZAP-S-myc; L, ZAP-L untagged. Significant changes were calculated using unpaired two-sided Student’s *t* tests; n.s., not significant; *P < 0.05; ***P < 0.001. Download FIG S2, TIF file, 0.4 MB. Copyright © 2021 Gonzalez-Perez et al. 2021 Gonzalez-Perez et al. https://creativecommons.org/licenses/by/4.0/ This content is distributed under the terms of the Creative Commons Attribution 4.0 International license.

Taken together, these findings show that both ZAP-S and ZAP-L negatively affect HCMV genome replication.

### ZAP negatively affects global expression of early and late HCMV proteins.

**FIG 3:**
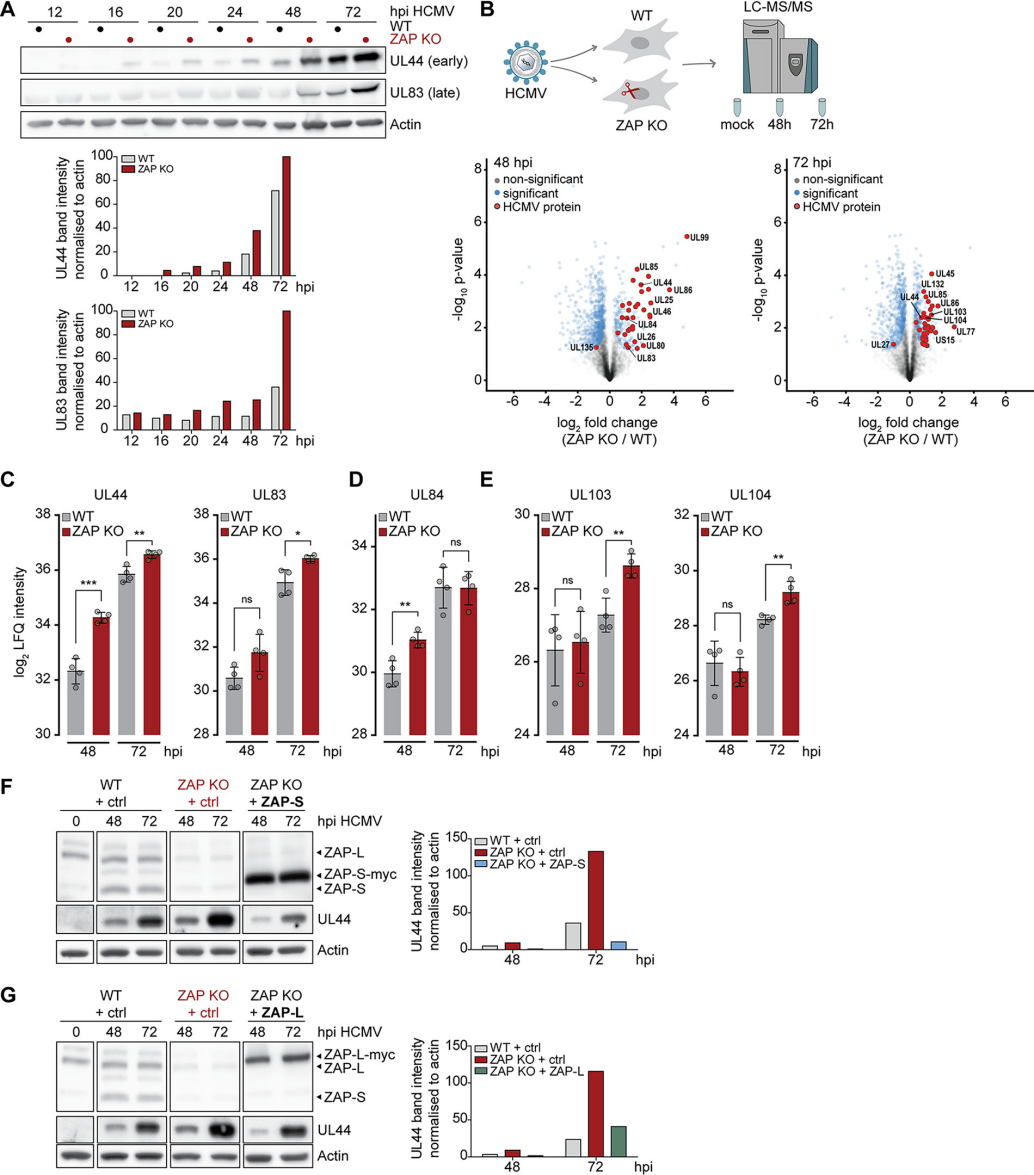
ZAP has a negative impact on early and late HCMV protein levels. (A) WT or ZAP KO HFF-1 cells were infected by centrifugal enhancement with HCMV (MOI 0.1), and lysates were analyzed at the indicated time points postinfection by immunoblotting with specific antibodies against HCMV UL44, HCMV UL83, and actin. One representative experiment performed with three independent ZAP KO cell lines is shown, with similar results in all three experiments. Quantifications of UL44 and UL83 band intensities normalized to actin are represented as bar plots. (B) WT and ZAP KO HFF-1 cells were mock-treated or infected by centrifugal enhancement with HCMV (MOI 0.1), and cell lysates were subjected to total proteome LC-MS/MS analysis at the indicated time points. Represented are volcano plots (*x* axis, log_2_ fold change; *y* axis, -log_10_ *P* value) showing differentially expressed proteins at 48 and 72 h post-HCMV infection (unpaired two-sided Student’s *t* test with permutation-based FDR, 0.05; S0, 0.1). (C to E) Time-resolved expression changes of HCMV UL44 and UL83 (C), UL84 (D), UL103, or UL104 (E) in HCMV-infected WT and ZAP KO HFF-1 cells displayed as bar plots showing the mean ± S.D. of quadruplicates. (F and G) WT, ZAP KO, or ZAP KO HFF-1 cells reconstituted with either ZAP-S (F) or ZAP-L (G) were infected by centrifugal enhancement with HCMV (MOI 0.1), and lysates were analyzed at the indicated time points postinfection by immunoblotting with specific antibodies against ZAP, HCMV UL44, and actin. Quantifications of UL44 band intensities normalized to actin are depicted as bar plots. One representative of at least 2 independent experiments is shown. hpi, hours postinfection. Significant changes were calculated using unpaired two-sided Student’s *t* tests; n.s., not significant; *, *P < *0.05; **, *P < *0.01; ***, *P < *0.001.

Given the negative impact of ZAP on HCMV genome replication, we next examined the time course of HCMV infection in the presence or absence of ZAP. Expression of HCMV genes follows a temporal cascade, which begins with the transcription of immediate-early (IE) genes, with the translation of IE proteins starting approximately 6 h postinfection (hpi). IE proteins subsequently transactivate the transcription of early (E) genes, which are mainly involved in viral DNA replication. Early proteins are produced approximately 18 to 20 hpi and together with a third classical cluster of the so-called early-late (E-L) proteins at 48 hpi will mediate the transcription of late (L) genes, which code mainly for viral capsid, envelope, and tegument components at 72 to 96 hpi (32–34). When we monitored the expression levels of the early viral protein UL44 and the late viral protein UL83 in HCMV-infected WT and the three ZAP KO cell lines, we observed elevated protein levels of UL44 and UL83 in the absence of ZAP. This suggests that the presence of ZAP negatively affects viral protein expression (Fig. 3A), which is in line with our analysis of HCMV genome replication (Fig. 2B).

To obtain a global overview of the progression of HCMV infection, we performed whole-proteome analyses of WT and ZAP KO HFF-1 cells using liquid chromatography with tandem mass spectrometry (LC-MS/MS) (Data set S1). We mock-treated or infected WT and ZAP KO HFF-1 cells with HCMV for 48 and 72 h, thus covering the early and late proteome landscape of viral gene expression. Overall, we observed significantly higher viral protein levels in ZAP KO cells than in WT cells (Fig. 3B). In line with our previous observations (Fig. 3A), we detected significantly higher levels of the early UL44 protein and the late UL83 protein in ZAP KO cells (Fig. 3C). Moreover, the proteome analysis showed other viral proteins that were significantly upregulated in the absence of ZAP. For instance, we detected increased expression of UL84, which is an early protein involved in viral DNA replication, at 48 hpi (Fig. 3D), as well as elevated levels of the late proteins UL103 and UL104 at 72 hpi, similar to UL83 and corresponding to late kinetics (Fig. 3E).

10.1128/mBio.02683-20.1 DATA SET S1 Proteome analyses. Download Data Set S1, XLSX file, 7.7 MB. Copyright © 2021 Gonzalez-Perez et al. 2021 Gonzalez-Perez et al. https://creativecommons.org/licenses/by/4.0/ This content is distributed under the terms of the Creative Commons Attribution 4.0 International license.

Next, we performed reconstitution assays with myc-tagged ZAP-S and ZAP-L as described above (Fig. 2C) and analyzed UL44 protein expression by immunoblotting (Fig. 3F and G). Similar to our analysis of HCMV genome replication, UL44 protein levels in ZAP KO cells reconstituted with either myc-tagged ZAP-S (Fig. 3F) or myc-tagged ZAP-L (Fig. 3G) were lower than in the absence of ZAP and comparable to those in WT cells. Similar results were obtained with untagged ZAP-L (Fig. S2B).

Taken together, these results demonstrate that the presence of both main ZAP isoforms negatively affects HCMV protein levels at early and late stages of infection.

### ZAP-S and ZAP-L have a negative impact on early and late HCMV transcripts.

**FIG 4:**
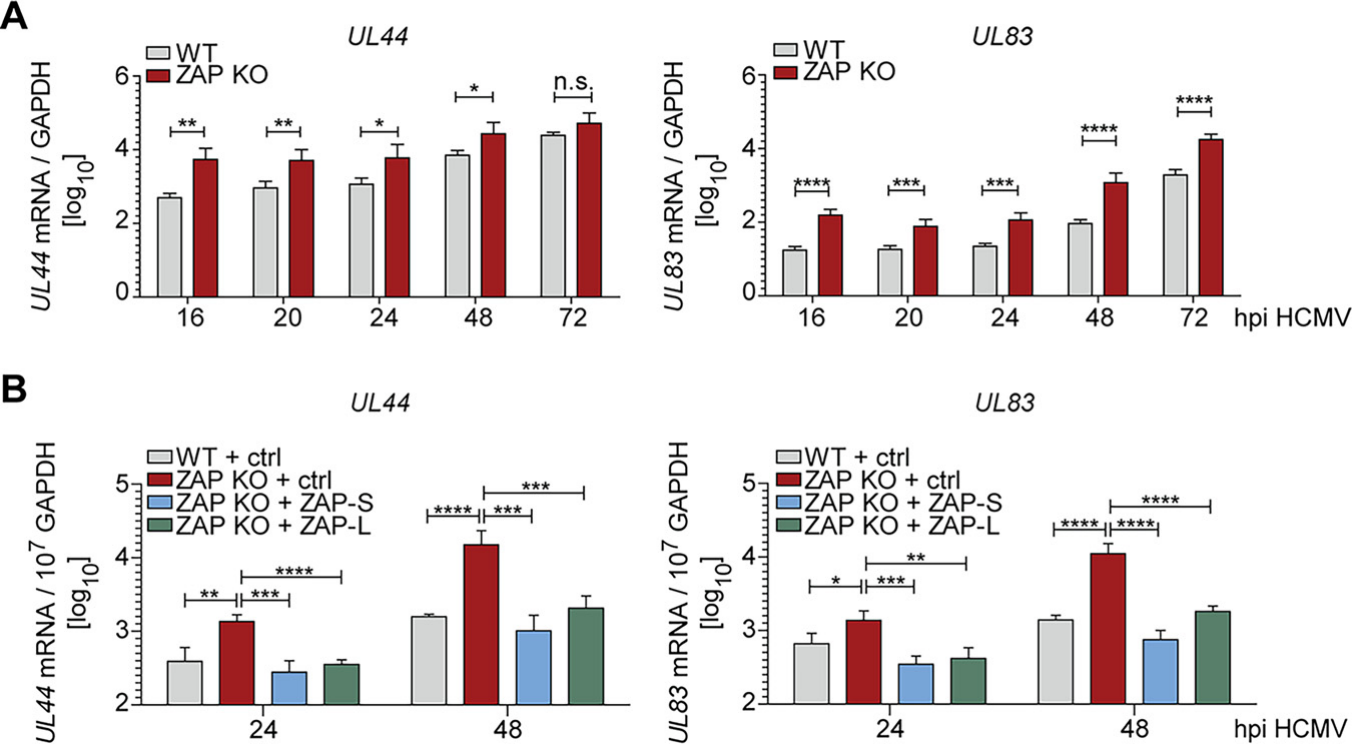
ZAP-S and ZAP-L negatively affect early and late HCMV transcripts. (A) WT or ZAP KO HFF-1 cells were infected by centrifugal enhancement with HCMV (MOI 0.1). Total RNA was extracted at the indicated time points postinfection, and mRNA levels of HCMV *UL44* and *UL83* were measured by qRT-PCR. Viral mRNA relative expression (log_10_) normalized to *GAPDH* is displayed as bar plots showing the mean ± S.D. of three independent experiments performed with experimental duplicates. Experiments were performed in three independent ZAP KO cell lines, and results were combined. (B) ZAP KO HFF-1 stably expressing either ZAP-S or ZAP-L or transduced with empty vector control, and WT cells expressing empty vector, were infected by centrifugal enhancement with HCMV (MOI 0.1) and qRT-PCR performed as described in panel A. Viral mRNA relative expression (log_10_) normalized to *GAPDH* is displayed as bar plots showing the mean ± S.D. of two independent experiments performed with experimental duplicates. hpi, hours postinfection. Significant changes were calculated using unpaired two-sided Student’s *t* tests; n.s. not significant; *, *P < *0.05; **, *P < *0.01; ***, *P < *0.001; ****, *P < *0.0001.

Previous studies showed that ZAP directly binds to RNA (14) and subsequently mediates its degradation by recruiting both the 5′and 3′ RNA degradation machinery (13, 16). To decipher whether ZAP affects HCMV mRNA expression, we analyzed mRNA levels of the early *UL44* and the late *UL83* transcripts at different stages of the HCMV infection cycle by quantitative reverse transcription-PCR (qRT-PCR) in WT and ZAP KO cells. Indeed, in the presence of ZAP, *UL44* and *UL83* mRNA levels were lower (Fig. 4A), which mirrors our protein analyses. Congruent with our analysis of HCMV protein expression (Fig. 3), reconstitution of ZAP KO cells with either ZAP-S or ZAP-L resulted in the rescue of this phenotype (Fig. 4B).

Taken together, these results suggest that both ZAP isoforms, ZAP-S and ZAP-L, negatively regulate HCMV mRNA expression, possibly by affecting either their expression or stability or by other, indirect effects.

### HCMV infection progresses faster in the absence of ZAP.

**FIG 5:**
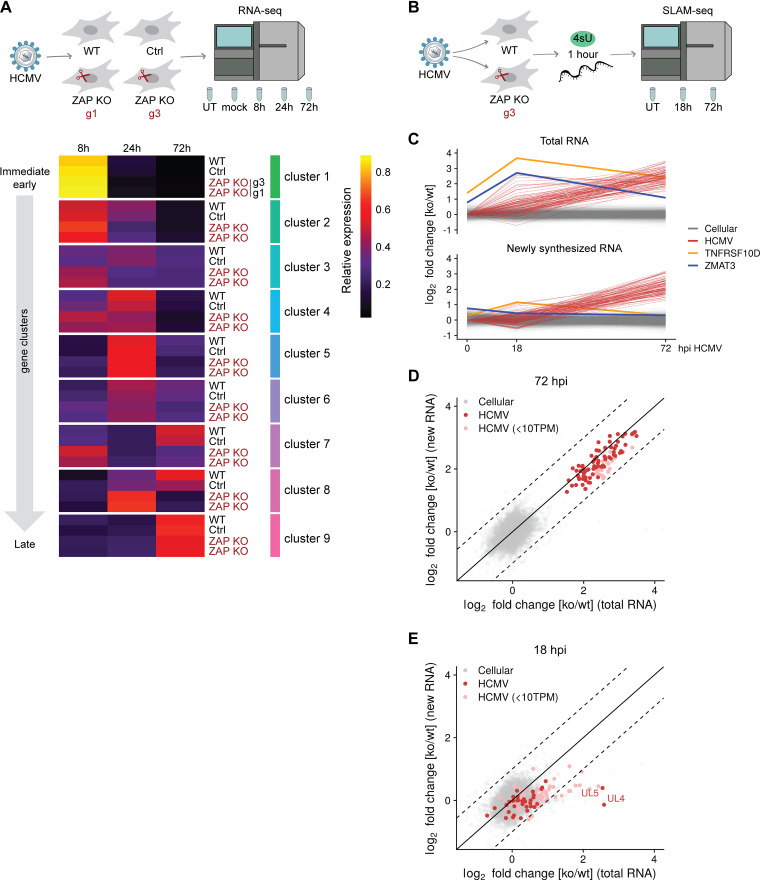
ZAP decelerates HCMV infection and negatively affects the stability of a subset of HCMV transcripts. (A) WT, control, and two independent ZAP KO HFF-1 cell lines were untreated (UT), mock-treated, or infected by centrifugal enhancement with HCMV (MOI 0.1). Total RNA was extracted at 8, 24, and 72 hpi, and lysates were subjected to total transcriptome analysis. Relative temporal expression levels of gene clusters and selected individual genes are represented as a heat map. Expression of HCMV genes was quantified from the RNA-sequencing and relative temporal expression levels calculated by dividing, per-sample, normalized expression values (fpkm) to the sum of these values from the same gene over all samples/time points. Based on these values, genes were clustered in nine groups representing kinetic classes. Shown are the averages of replicates and clusters. g1, g3, ZAP KO generated with gRNA 1 or gRNA 3. (B) WT and ZAP KO HFF-1 cells were left untreated (UT) or infected by centrifugal enhancement with HCMV (MOI 0.1). Newly synthesized RNA was labeled with 4-thiouridine (4sU) for 1 h prior to cell lysis, and lysates were taken at 18 and 72 hpi, followed by RNA purification. SLAM-seq was performed to identify newly synthesized and total RNA using GRAND-SLAM. (C) Time courses of log_2_ fold changes of cellular and viral genes (*n* = 12,652) for total (upper panel) and newly synthesized (lower panel) RNA in ZAP KO/WT HFF-1 cells. The values represent the mean of two biological replicates. *TNFRSF10D* and *ZMAT3* are indicated in yellow and purple, respectively. (D and E) Represented are log_2_ fold changes of cellular and viral genes (*n* = 12,652) for total (*x* axis) and newly synthesized RNA (*y* axis) at 72 (D) and 18 hpi (E). Values represent the mean of two biological replicates. Weakly expressed viral genes are indicated (<10 TPM). Dashed lines demarcate 2-fold differences in regulation of total versus newly synthesized RNA. hpi, hours postinfection; TPM, transcripts per million.

In order to understand the effect of ZAP on the viral gene expression cascade over time, we analyzed the whole-transcriptome landscape during HCMV infection by RNA-sequencing (RNA-seq) in WT, control cells, and two independent ZAP KO cell lines (g1 and g3 ZAP KO) (Fig. 5A, Data set S2). First, we observed that HCMV infection leads to a robust and overall similar induction of ISGs in all cell lines analyzed, indicating that the absence of ZAP does not affect the PRR-mediated host response during HCMV infection (Fig. S3). For analysis of the temporal progression of HCMV gene expression, we focused on the mRNA abundance per time point postinfection relative to the abundance over all time points. Based on these relative temporal gene expression values in WT cells, genes were clustered into nine groups grouped from immediate-early to late expression, broadly corresponding to a previously published classification (32) (Fig. 5A, Fig. S4). To simplify the temporal distribution of the viral genes, average relative expression levels of the genes within the clusters were calculated and depicted as a heat map (Fig. 5A). Strikingly, the temporal expression patterns of most clusters were shifted to earlier time points in both ZAP KO cell lines compared to WT and control cells. For instance, ZAP KO cells showed high expression levels of *UL138* or long noncoding *RNA 2.7* (Fig. S4). Notably, these viral transcripts have been previously identified in several studies in relation to the latent phase of HCMV (35–37), a concept that is currently changing and is increasingly associated with a late-lytic replication program (38). Although the effect of earlier expression patterns in ZAP KO cells was not uniform, it was particularly strong for cluster 7, which contains the genes *UL141*, *US27*, and *US28*, and for cluster 8 (*UL4*, *UL5*, *UL6*, *UL148A*).

10.1128/mBio.02683-20.2 DATA SET S2 Transcriptome analyses (RNA-seq). Download Data Set S2, XLSX file, 12.7 MB. Copyright © 2021 Gonzalez-Perez et al. 2021 Gonzalez-Perez et al. https://creativecommons.org/licenses/by/4.0/ This content is distributed under the terms of the Creative Commons Attribution 4.0 International license.

10.1128/mBio.02683-20.7 FIG S3 ISG expression is similar in HCMV-infected WT and ZAP KO cells. WT and ZAP KO HFF-1 cells were untreated or infected by centrifugal enhancement with HCMV (MOI 0.1). Total RNA was extracted at 24 hpi, and lysates were subjected to total transcriptome analysis. Represented are log_2_ transformed fold changes at 24 hpi compared to untreated cells of WT and ZAP KO (g3) cell lines, calculated using edgeR and plotted against each other. ISGs are depicted in red. hpi, hours postinfection. Download FIG S3, TIF file, 0.1 MB. Copyright © 2021 Gonzalez-Perez et al. 2021 Gonzalez-Perez et al. https://creativecommons.org/licenses/by/4.0/ This content is distributed under the terms of the Creative Commons Attribution 4.0 International license.

10.1128/mBio.02683-20.8 FIG S4 Relative temporal expression levels of HCMV genes above a reasonable expression threshold at 8, 24, and 72 hpi. WT, control, and two independent ZAP KO HFF-1 cell lines were left untreated, mock-treated, or infected by centrifugal enhancement with HCMV (MOI 0.1). Total RNA was extracted at 8, 24, and 72 hpi, and lysates were subjected to total transcriptome analysis. Expression of HCMV genes was quantified from the RNA-sequencing analysis and relative temporal expression levels calculated by dividing per-sample normalized expression values (fpkm) to the sum of these values from the same gene over six samples of the same cell line. Based on these values, genes were grouped using unsupervised clustering, and the clusters, representing kinetic classes, were ordered from immediate-early (top) to late (bottom). In addition, shown to the right is the kinetic classification from Weekes et al. (32) (immediate-early, early, late), where available, or if the gene codes for a noncoding RNA. UL138 and RNA2.7 are marked with an asterisk (*). Download FIG S4, TIF file, 1.8 MB. Copyright © 2021 Gonzalez-Perez et al. 2021 Gonzalez-Perez et al. https://creativecommons.org/licenses/by/4.0/ This content is distributed under the terms of the Creative Commons Attribution 4.0 International license.

In conclusion, ZAP KO cells show an accelerated course of HCMV infection and seem to more rapidly achieve an intracellular environment associated with a late-lytic gene expression program. These results, in line with our findings for viral protein levels, indicate that ZAP delays the progression of the HCMV infection cycle, as reflected in the decelerated course of the viral gene expression cascade.

### ZAP affects the stability of early, but not late, HCMV transcripts.

ZAP was previously described to be involved in mRNA degradation. To investigate the mechanisms by which ZAP interferes with HCMV infection, we analyzed cellular and viral mRNA stability during HCMV infection of WT and ZAP KO cells. For this, we labeled newly synthesized RNA at 17 h or 71 h postinfection for 1 h with 4-thiouridine (4sU), as well as control untreated cells, and performed SLAM-seq (thiol-linked alkylation for the metabolic sequencing of RNA) (39). Then, we identified the newly synthesized and total RNA using the computational approach GRAND-SLAM (termed globally refined analysis of newly transcribed RNA and decay rates using SLAM-seq) (40) (Fig. 5B, Data set S3). In agreement with previous studies, we confirmed that *TNFRSF10D* total mRNA was significantly upregulated in uninfected ZAP KO cells but also expressed higher in the context of HCMV infection (Fig. 5C). *TNFRSF10D* encodes the prosurvival protein TRAIL receptor 4 (TRAILR4, a human cell surface receptor of the TNF-receptor superfamily) and was previously described to be targeted by ZAP at the mRNA level (41). Notably, we found another, previously undescribed, antiapoptotic factor which was significantly upregulated in ZAP KO cells compared to WT cells, *ZMAT3* (encoding the zinc finger matrin-type protein 3, also known as zinc finger protein Wig-1), with a more pronounced upregulation in the context of HCMV infection (Fig. 5C, Fig. S5). For both *TNFRSF10D* and *ZMAT3*, reconstitution with either ZAP-S or ZAP-L rescued these phenotypes as shown by qRT-PCR analyses (Fig. S5). The SLAM-seq results revealed that the upregulation in ZAP KO cells of these two cellular mRNAs in total RNA was not paralleled by the transcription of newly synthesized RNA, where levels were equal between WT and ZAP KO cells (Fig. 5C). This shows that in the absence of ZAP, these transcripts have a longer half-life and indicates that ZAP has an impact on their degradation, but not on their transcription.

10.1128/mBio.02683-20.3 DATA SET S3 SLAM-sequencing. Download Data Set S3, XLSX file, 4.2 MB. Copyright © 2021 Gonzalez-Perez et al. 2021 Gonzalez-Perez et al. https://creativecommons.org/licenses/by/4.0/ This content is distributed under the terms of the Creative Commons Attribution 4.0 International license.

10.1128/mBio.02683-20.9 FIG S5 The presence of ZAP-S and ZAP-L leads to reduced *TNFRSF10D* and *ZMAT3* cellular transcripts levels. WT, ZAP KO, and ZAP KO HFF-1 cells expressing either ZAP-S (blue) or ZAP-L (green) were mock-treated or infected by centrifugal enhancement with HCMV (MOI 0.1). At 24 hpi, total RNA was extracted, and qRT-PCR for *TNFRSF10D* and *ZMAT3* mRNA was performed. Cellular mRNA expression normalized to *GAPDH* is displayed as bar plots showing the mean ± S.D. of experimental duplicates. One representative of two independent experiments is shown. hpi, hours postinfection. Download FIG S5, TIF file, 0.2 MB. Copyright © 2021 Gonzalez-Perez et al. 2021 Gonzalez-Perez et al. https://creativecommons.org/licenses/by/4.0/ This content is distributed under the terms of the Creative Commons Attribution 4.0 International license.

For viral mRNA transcripts, we observed a 4- to 12-fold upregulation in ZAP KO cells on both total RNA as well as newly synthesized RNA levels at 72 hpi (Fig. 5D). These results indicate that the upregulation on total RNA levels in ZAP KO cells at late times of HCMV infection is predominantly due to increased transcription rates and not due to an increase in mRNA stability.

At 18 hpi, upregulation of viral transcripts was much weaker and below 2-fold for genes with significant levels of expression (>10 transcripts per million, TPM), with the exception of *UL4* and *UL5*, which were 6-fold upregulated (Fig. 5E). Strikingly, the upregulation in total RNA was not paralleled by upregulation of newly synthesized RNA. This provides evidence that at 18 hpi, a small subset of viral transcripts, including *UL4* and *UL5*, is destabilized in the presence of ZAP. We conclude that ZAP directly interferes with the expression of distinct viral genes early during infection on the posttranscriptional level, which, as a secondary effect, results in substantially weaker transcription of viral genes at later time points.

### ZAP binds to cytosine-rich regions in several thousand cellular mRNAs.

**FIG 6:**
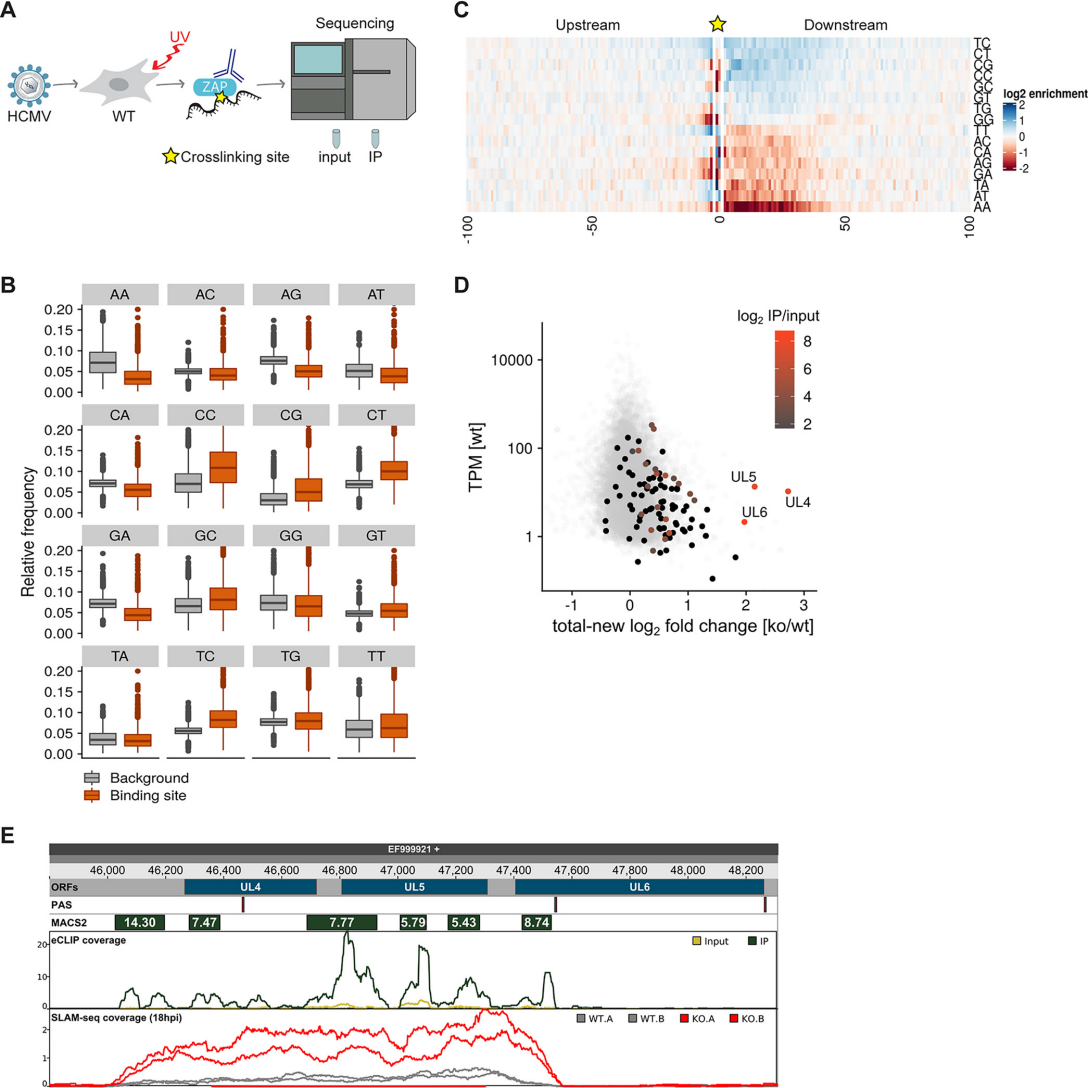
ZAP specifically binds to a subset of cytosine-rich viral transcripts early during HCMV infection. (A) WT HFF-1 cells were infected with HCMV (MOI 1) and UV-cross-linked 20 hpi to create covalent bonds between nucleic acids and associated proteins. Following cell lysis, unprotected RNA was digested and endogenous ZAP was immunoprecipitated using a ZAP-specific antibody. Cross-linked protein-RNA complexes were separated by SDS-PAGE, followed by transfer onto nitrocellulose. Bands migrating at the expected size range were excised, and recovered RNA fragments were converted into a cDNA library. IP samples and size-matched input were subjected to sequencing according to the eCLIP protocol. The cross-linking site is depicted as a star. (B) Boxplots showing the distribution of relative frequencies of dinucleotides inside (“binding site”) and outside (“background”) binding sites for *n* = 6,600 strongly expressed genes (TPM > 10). The *y* axis is cut at 0.2. (C) Heat map showing the log_2_ enrichments of dinucleotide frequencies over background at each position upstream and downstream of the main cross-linking site per gene (*n* = 6,600). (D) Scatterplot comparing the deviation of KO versus WT fold changes in newly synthesized RNA from total RNA against the total expression level in WT at 18 h postinfection (hpi). Viral open reading frames (ORFs) are colored according to their strongest binding site (in terms of enrichment over input RNA) or shown in black if no binding site was detected. Cellular genes are shown in gray for comparison. TPM, transcripts per million. (E) Genome browser showing the *UL4-UL6* locus. The tracks indicate the position in the HCMV genome, the ORF positions, polyadenylation signals (PAS), binding sites called by MACS2 with the indicated enrichment values, the normalized coverage of eCLIP reads (input in ochre, IP in green), and the normalized coverage by SLAM-seq reads at 18 h postinfection (ZAP KO cells in red, WT cells in gray).

Previous studies indicated that ZAP directly binds to CG-dinucleotide-enriched RNA sequences (19, 29). To determine the physical mRNA binding sites of ZAP during HCMV infection, we performed enhanced cross-linking and immunoprecipitation in combination with RNA sequencing analysis (eCLIP-seq) (42) using a ZAP antibody that recognizes both ZAP isoforms (Fig. 6A). We first concentrated on the host genome where we found 15,302 ZAP binding sites in 5,657 transcripts (Data set S4). To assess the quality of our data, we compared the expression changes of cellular mRNAs with and without ZAP binding sites in WT and ZAP KO cells. For that, we focused on the untreated samples from our SLAM-seq experiment to exclude effects of ZAP that are not mediated by direct interaction with targets, which likely are exacerbated upon infection. Gene expression differences were generally weak, with >95% of genes regulated less than 1.3-fold. Nevertheless, genes with a strong ZAP binding site (>5-fold enrichment of size-matched input RNA) or with multiple binding sites had on average higher expression levels in KO cells (*P* < 6.4 × 10^−6^, two-sided Kolmogorov-Smirnov test.) (Figure S6A). In contrast, newly synthesized RNA was largely unchanged (Fig. S6B). This shows that even in uninfected cells, ZAP has a measurable impact on RNA half-lives of hundreds of cellular genes and that our eCLIP approach could capture the global landscape of ZAP binding sites. For genes with a single weak binding site of ZAP (<5-fold enrichment over input RNA), we did not observe an effect on expression levels (Fig. S6A). Weak binding sites might correspond to transient ZAP binding without a strong effect on RNA stability, or they are predominantly false-positive peak calls. Thus, we concentrated on strong ZAP binding sites in the following analyses.

10.1128/mBio.02683-20.4 DATA SET S4 eCLIP-sequencing. Download Data Set S4, XLSX file, 2.5 MB. Copyright © 2021 Gonzalez-Perez et al. 2021 Gonzalez-Perez et al. https://creativecommons.org/licenses/by/4.0/ This content is distributed under the terms of the Creative Commons Attribution 4.0 International license.

10.1128/mBio.02683-20.10 FIG S6 ZAP binds to cellular transcripts with no apparent specific motif. (A and B) Empirical cumulative distributions of log_2_ fold changes comparing KO and WT in total RNA (A) or newly synthesized RNA (B) in untreated samples are shown. Genes are stratified according to the number of detected ZAP binding sites in eCLIP data (“weak” binding sites are defined as having <5× enrichment over input RNA). *P* values are from a two-sided Kolmogorov-Smirnov test comparing against genes without binding sites, and the numbers of genes per stratum are indicated. (C) YTTCC motif identified by DREME in 1,268 out of 2,158 binding sites 1 to 50 nt downstream of the main cross-linking site and in 729 out of 2,158 shuffled control sequences. (D) AGRA motif identified by DREME in 990 out of 2,158 binding sites 1 to 50 nt downstream of the main cross-linking site and in 698 out of 2,158 shuffled control sequences. (E) GCYGCYGC motif identified by DREME in 269 out of 2,158 binding sites 1 to 50 nt downstream of the main cross-linking site and in 83 out of 2,158 shuffled control sequences. Download FIG S6, TIF file, 0.5 MB. Copyright © 2021 Gonzalez-Perez et al. 2021 Gonzalez-Perez et al. https://creativecommons.org/licenses/by/4.0/ This content is distributed under the terms of the Creative Commons Attribution 4.0 International license.

To evaluate the binding preferences of ZAP in our data, we counted dinucleotides inside or outside binding sites in strongly expressed genes (TPM > 10, *n* = 6,600) at 18 hpi. As observed in previous studies, CG dinucleotides in ZAP binding sites were significantly overrepresented (enrichment over background *f* = 1.65, *P* < 2 × 10^−16^, two-sided Wilcoxon test). However, we also observed other cytosine-containing dinucleotides, including CC (*f* = 1.55, *P* < 2 × 10^−16^), TC (*f* = 1.48, *P* < 2 × 10^−16^), and CT (*f* = 1.46, *P* < 2 × 10^−16^), to be strongly enriched in binding sites. In contrast, adenosine-containing dinucleotides, with the exception of TA, were generally underrepresented (AA, *f* = 0.46; GA, *f* = 0.62; AG, *f* = 0.67; AT, *f* = 0.75; CA, *f* = 0.78; AC, *f* = 0.80; all, *P* < 2 × 10^−16^; Fig. 6B).

To investigate the binding preferences of ZAP in more detail, we made use of the nucleotide resolution of our eCLIP data; for each gene with a detected binding site, we identified the strongest cross-linking site by considering the 3′ ends of mapped eCLIP read pairs and counted occurrences of dinucleotides around these sites. This more detailed analysis confirmed the over- and underrepresentation of specific dinucleotides. Moreover, it revealed much stronger enrichments of cytosine-containing dinucleotides at specific positions downstream of the cross-linking sites than predicted by the prior analysis (CG, *f* = 2.08; CC, *f* = 2.00; TC, *f* = 1.77; CT, *f* = 1.67) and much stronger depletion of adenosine-containing dinucleotides (CA, *f* = 0.13; AA, *f* = 0.20; AT, *f* = 0.31; AC, *f* = 0.51; GA, *f* = 0.51; AG, *f* = 0.53; Fig. 6C). We then used DREME (43) to identify motifs enriched in the sequences directly downstream of the main cross-linking site. However, only a few spurious motifs were identified (Fig. S6C to E). As these were largely composed of overrepresented dinucleotides, we concluded that ZAP preferentially binds to cytosine-rich regions devoid of adenosines but likely does not recognize longer sequence patterns.

### ZAP binds to a limited number of sites in the viral transcriptome to destabilize mRNAs transcribed from the *UL4-UL6* locus.

Next, we identified ZAP binding sites in the viral transcriptome and mapped them to open reading frames (ORFs). Interestingly, we found only 16 binding sites exceeding an enrichment of >5-fold over input, 12 of which could be mapped to an ORF of the HCMV genome (Data set S4). Of note, five of those mapped to the *UL4-UL6* locus. Thus, ZAP binds to only a small number of sites on viral mRNAs, and those are concentrated in the *UL4-UL6* locus.

We then aimed to directly compare the ZAP binding sites identified by eCLIP with the regulation at 18 hpi. For that, we plotted the log fold change difference in total versus newly synthesized RNA (i.e., the deviation from the diagonal line in Fig. 5E) against the overall expression strength in the WT and determined for each ORF the most strongly enriched binding site (Fig. 6D). As shown before, transcripts expressed from the *UL4-UL6* locus were by far the most strongly destabilized mRNAs (Fig. 5E), with a strong temporal shift upon ZAP depletion (Fig. 5A, Fig. S4, cluster 8). Strikingly, our analysis revealed that these transcripts are also the only direct targets of ZAP with a substantial enrichment over input as shown by eCLIP-seq (Fig. 6E). Our SLAM-seq data indicate that transcription at this locus stops at a polyadenylation signal inside the UL6 ORF and that a sixth binding site upstream of the UL4 ORF that was not considered in the previous analysis is also located on a transcript expressed from this locus (Fig. 6E).

**FIG 7:**
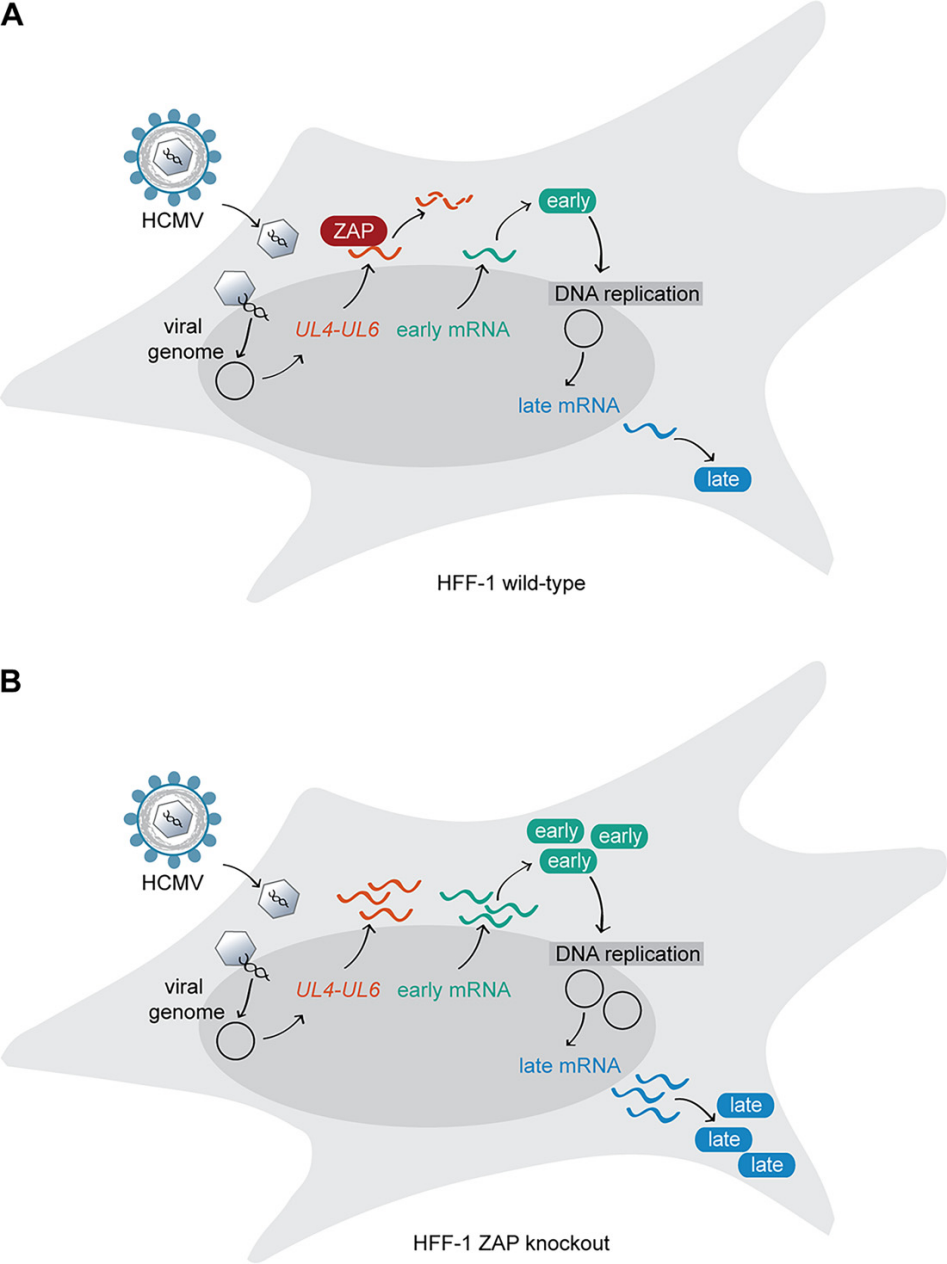
ZAP is a restriction factor for HCMV infection. (A) The expression of the ISG ZAP is induced at early stages during HCMV infection. The RNA-binding protein ZAP specifically binds to viral mRNAs transcribed from the HCMV *UL4-UL6* genome locus (depicted in orange) and negatively affects their stability. In the presence of ZAP, expression of early and late HCMV genes and proteins, as well as genome replication, is delayed. (B) In the absence of ZAP, the stability of HCMV mRNAs transcribed from the *UL4-UL6* locus is unaltered, and HCMV transcription, protein expression, and genome replication are enhanced compared to ZAP-expressing cells.

Altogether, our data provide clear evidence that ZAP strongly destabilizes mRNAs originating from the UL4 and UL5 ORFs by directly binding to several mRNA sites and that these are the most prominent ZAP targets among the transcripts produced from the HCMV genome. We therefore propose that this early and distinct regulation of the *UL4-UL6* locus early after HCMV infection could underlie the observed delay in the progression of the HCMV infection cycle (Fig. 7).

## DISCUSSION

Viral infection induces a specific expression pattern of ISGs (44). To date, more than 300 ISGs have been described, but so far, the function of the majority of the proteins they encode is poorly understood. ISG function is highly contextual, as their effect on viral infection is dependent on the viral entry route, replication mechanism, site of replication and viral assembly, and cell type. Hence, while some ISGs may exert an antiviral activity against some viruses, they may either have a neutral or positive effect on other viruses or, in some instances, be susceptible to viral evasion mechanisms (5, 7).

HCMV infection leads to the upregulation of a distinct set of cellular proteins during the first 24 h of infection, 32 of which were classified as ISGs, including the RNA binding protein ZAP (32). While that report did not distinguish between the two major ZAP isoforms, ZAP-S and ZAP-L, in this study, we delineated their endogenous expression kinetics during HCMV infection. ZAP-L is readily detectable in uninfected cells, and its expression slightly increases throughout the first 48 h of HCMV infection, while ZAP-S protein levels are low in uninfected cells and strongly upregulated from 6 h postinfection onward. At a late stage of the HCMV life cycle, expression of both ZAP isoforms decreases, which likely reflects the fading type I IFN response rather than HCMV-mediated degradation (32, 45).

Our results showed that HCMV replication is restricted by the presence of ZAP. Notably, reconstitution of ZAP KO cells with either ZAP-S or ZAP-L showed that both have the potential to restrict HCMV replication to similar levels as endogenous ZAP in wild-type cells. However, the overexpression of one or another ZAP isoform during reconstitution did not push antiviral protection beyond that of endogenous levels. We speculate that this could (i) be a phenomenon of saturation regarding the capacity of cellular machineries to degrade RNA or (ii) be related to the availability of cofactors involved in ZAP-mediated antiviral activities (46–48).

While both ZAP isoforms, ZAP-S and ZAP-L, restricted HCMV infection in our study, recent studies propose diverse functions for one or the other isoform during infection with different viruses, for example, the DNA virus modified vaccinia virus Ankara (MVA) (27) and Sindbis virus (SINV), an RNA alphavirus (17, 31). For MVA, which replicates in the cytoplasm, no impact of ZAP on viral transcription was observed, but rather, an effect of ZAP-L on viral assembly was observed (27). Similarly, Schwerk and colleagues found that ZAP-L, which can be farnesylated at its C terminus (lacking in ZAP-S) and is thereby targeted to membranes (10), colocalizes with SINV RNA intermediates in distinct foci in the cytoplasm (31). Moreover, ZAP-S was described to act as a negative feedback regulator of the IFN response later in infection with SINV by destabilizing *IFN* transcripts (31). However, in our study, by comparing WT and ZAP KO cells, we did not observe an effect of ZAP expression on IFN signaling pathways. This could be due to the presence of HCMV evasion proteins that downmodulate the IFN response, which may overshadow an effect of ZAP-S in this regard (7, 49), but this is purely speculative at this point. Nevertheless, based on our results, we cannot state whether ZAP-S and ZAP-L employ the same mechanism of action or whether they provide individual contributions to inhibit HCMV replication. This will need to be further evaluated in future work.

We took a global approach to explore the role of ZAP during HCMV infection and examined the whole transcriptome and proteome in WT and ZAP KO cells. We observed that expression of ZAP greatly delays transcription of the majority of viral genes, eventually resulting in a delay of viral protein expression. CCCH-type zinc finger proteins such as ZAP have previously been reported to be capable of RNA binding and mediation of RNA degradation (13, 14, 16). In the case of ZAP, a preference for RNA with a high CG dinucleotide content was proposed (19, 20, 30). In comparison to the human genome, which has low CG content (50, 51), the HCMV genome presents the highest CG content among human *Betaherpesvirinae* (52), which makes HCMV transcripts a putative target for ZAP-mediated degradation. Accordingly, we conducted a series of experiments with regard to mRNA stability during HCMV infection. By performing eCLIP-seq and SLAM-seq, we investigated whether (i) ZAP binds to cellular and/or viral mRNA, (ii) ZAP binds to specific mRNA sequences, and (iii) the stability of the ZAP-bound mRNA is altered.

First, due to the nucleotide resolution of eCLIP-seq, dinucleotide analyses carried out on the strongest cross-linking sites enabled the identification of putative binding preferences for ZAP. The obtained results were in agreement with previous publications that show a binding preference of ZAP to CG-rich motifs (19, 20, 29, 30, 53). However, our analysis revealed that not only CG, but also other cytosine-containing dinucleotides were highly overrepresented in the cross-linking sites, whereas adenosines were significantly underrepresented. Ultimately, our data did not provide specific sequence motifs for ZAP binding, but only few spurious motifs were identified. As these were largely composed of overrepresented dinucleotides, we conclude that ZAP likely does not recognize longer specific sequence patterns but preferentially binds to cytosine-rich regions. At this point, we can only hypothesize that there may also be (i) sequences surrounding the ZAP binding sites or (ii) secondary RNA structures that are crucial for ZAP binding, as previously proposed by Huang et al. (28). These results also suggest a broader binding preference of ZAP to mRNA, which in turn, possibly allows ZAP to act against a variety of viruses, and virus-mediated escape of recognition by ZAP, e.g., by single nucleotide mutations, is less likely.

In agreement with previous studies, which described ZAP to mediate degradation not only of foreign but also of specific cellular transcripts (31, 41), we identified thousands of ZAP-bound cellular transcripts by eCLIP-seq. These transcripts were weakly but significantly downregulated in uninfected WT cells, indicating that ZAP, even when expressed at basal levels in interferon-naive cells, exerts a function as an mRNA destabilizer. Upon HCMV infection, several cellular mRNAs were strongly destabilized by ZAP. For example, we confirmed a previously reported negative effect of ZAP on *TNFRSF10D* (41), encoding the TRAIL receptor 4 (TRAILR4) involved in cell survival. The authors showed that ZAP-mediated downregulation of TRAILR4 results in increased cell sensitivity to TRAIL-mediated apoptosis (41). Moreover, our global approach allowed us to identify previously undescribed cellular targets of ZAP, as illustrated by the *ZMAT3* gene, encoding the zinc finger matrin-type protein 3 (also known as zinc finger protein Wig-1), which is another prosurvival factor (54).

As mentioned above, the HCMV genome has a high CG content compared to the host genome. However, expression of ZAP did not affect stability of viral transcripts expressed late in infection (72 hpi), but it affected viral transcripts expressed at early stages of infection (18 hpi), with HCMV *UL4* and *UL5* transcripts being the most prominent ones. Remarkably, eCLIP-seq revealed that most of the binding sites for ZAP were concentrated in the *UL4-UL6* locus. This strongly suggests that the overall CG content is not predictive for ZAP binding.

In the course of completing our manuscript, a study was published that reported a negative effect of ZAP on HCMV (25). The authors concluded, based on a bioinformatics analysis of the CG content of HCMV genes, that the low CG content of the *IE1* gene could be an HCMV evasion mechanism to avoid ZAP recognition (25). While the conclusion drawn by Lin et al. (25) is reasonably supported by their data, the impact of ZAP was only elucidated on four HCMV proteins, without analyzing their transcript levels or mRNA stability. Our study generally confirmed the results of Lin et al. (25), as we did observe a ZAP-dependent notable delay of viral protein expression but no effect on total *IE1* transcript levels. However, rather than IE1 expression being the decisive factor for this phenotype, our data suggest that the delay in HCMV life cycle progression is due to the effect of ZAP on the stability of transcripts from the *UL4-UL6* gene locus.

*UL4*, *UL5*, and *UL6* belong to the RL11 gene family of HCMV (55, 56). The *UL4* gene encodes two transcripts with early kinetics and one with late kinetics (57, 58), while *UL5* encodes two transcripts (59, 60). Interestingly, all of them share the same 3′ end, which designates the *UL4-UL5* region as a transcription unit (60). To our knowledge, *UL6* was not further characterized yet. The biological function of the RL11 family members is only poorly understood. Several studies suggest that these membrane-associated proteins may be involved in immune evasion (61–65), as they are largely dispensable for virus growth in cultured fibroblasts (66, 67). A more detailed impact of UL4 and UL6 on HCMV infection has not been studied so far. Recently, HCMV UL5 was shown to be involved in efficient viral assembly and/or egress from the host cell by interacting with the cellular scaffold protein IQGAP1 (IQ motif-containing GTPase activating protein 1) (68). This indicates a possible role of UL5 in a late phase of HCMV infection. However, the impact of *UL4* and *UL5* expressed with early kinetics remains unknown. The fact that ZAP has an impact on HCMV infection and specifically targets transcripts from this locus suggests a possible involvement of products originating from the *UL4-UL6* gene locus for efficient HCMV infection.

Altogether, these findings show the multiple layers of complexity of the RNA binding protein ZAP, highlighting its different facets depending on the virus species it encounters. For HCMV, ZAP appears on the scene at early time points of infection, decelerating the viral gene expression cascade, presumably by handpicking a distinct set of viral transcripts for degradation, mainly containing the UL4 and UL5 HCMV ORFs. Our study illustrates the potent role of ZAP as an antiviral restriction factor and sheds light on a possible role of UL4 and/or UL5 early during infection, paving a new avenue to explore these poorly characterized HCMV genes.

## MATERIALS AND METHODS

### Cell lines.

Primary human foreskin fibroblasts (HFF-1; SCRC-1041), MRC-5 (CCL-171), and human embryonic kidney 293T cells (HEK 293T; CRL‐3216) were obtained from ATCC. HEK 293T and MRC-5 cells were maintained in Dulbecco’s modified Eagle’s medium (DMEM; high glucose) supplemented with 8% fetal calf serum (FCS) and 1% penicillin/streptomycin (P/S). HFF-1 cells were maintained in DMEM (high glucose) supplemented with 15% FCS, 1% P/S and 1% nonessential amino acids (NEAA). Cells were cultured at 37°C in a humidified 7.5% CO_2_ incubator.

### Viruses.

The wild-type HCMV TB40-BAC4 (here designated HCMV WT) was characterized previously (69) and kindly provided by Martin Messerle (Institute of Virology, Hannover Medical School, Germany). HCMV BACs were reconstituted after transfection of MRC5 cells with purified BAC DNA. Reconstituted virus was propagated in HFF-1 cells, and virus was purified on a 10% Nycodenz cushion. The resulting virus pellets were resuspended in virus standard buffer (50 mM Tris-HCl pH 7.8, 12 mM KCl, 5 mM EDTA) and stored at −70°C. Infectious titer was determined by standard plaque assay and IE1 labeling using HFF-1 cells.

### Plasmids.

Expression plasmids for firefly luciferase (FFLuc, control) and ZAP-S (short isoform of ZAP) in pTRIP-IRES-RFP as well as pCMV-VSV-G and pCMV-gag/pol plasmids were described previously (44) and kindly provided by John Schoggins (University of Texas Southwestern Medical Center, Dallas, Texas). pcDNA4-HA-ZAP-L (long isoform of ZAP) (9) was kindly provided by Chad Swanson (Department of Infectious Diseases, School of Immunology and Microbial Sciences, King’s College London). ZAP-S and ZAP-L were subcloned into pEF1-V5/His (Thermo Fisher Scientific) via the KpnI/XbaI sites to generate pEF1-ZAP-S-V5/His and pEF1-ZAP-L-V5/His, respectively. Exchange of V5/His to myc/His was performed using the Q5 site-directed mutagenesis kit (New England Biolabs [NEB] no. E0554) according to the manufacturer’s protocol, resulting in pEF1-ZAP-S-myc/His and pEF1-ZAP-L-myc/His. In order to reconstitute ZAP-S and ZAP-L expression in ZAP KO cell lines, codon optimization of the ZAP-S and ZAP-L coding region was performed to prevent binding of the constitutively expressed gRNA and Cas9. For this, nucleotides 103 to 219 of the ZAP-S and ZAP-L coding region (spanning the binding sites for gRNA 1 and gRNA 3; see Fig. 1C) were codon optimized using the Q5 site-directed mutagenesis kit, resulting in pEF1-ZAP-S-myc/His and pEF1-ZAP-L-myc/His codon-optimized. A pTRIP-IRES-RFP empty vector was generated by replacing the coding region of ZAP-S from pTRIP-IRES-RFP ZAP-S (received from John Schoggins) by the multiple cloning sites of pWPI vectors to obtain PmeI, SdaI, SgsI, BamHI, XmaI, RgaI, and XhoI restriction sites for further subcloning. Codon-optimized versions of ZAP-S and ZAP-L were subcloned into the newly generated pTRIP-IRES-RFP empty vector via the SgsI/BamHI restriction sites to generate pTRIP-IRES-RFP ZAP-S-opt-myc/His and pTRIP-IRES-RFP ZAP-L-opt-myc/His. To generate the untagged version of ZAP-L, ZAP-L optimized was subcloned into pTRIP-IRES-RFP empty vector via the PmeI/BamHI sites, resulting in pTRIP-IRES-RFP ZAP-L-opt. All constructs were verified by sequencing. Oligo sequences as well as sequences of all constructs are available upon request.

The expression plasmid for gRNA cloning and CRISPR/Cas9-mediated gene editing, pLK05.U6.sgRNA(BsmBI,stuffer).EFS.SpCas9.P2A.tagRFP (70), was kindly provided by Dirk Heckl (Experimental Pediatrics, Martin Luther University, Halle, Germany). The corresponding envelope and packaging plasmids, pMD2.G and psPAX2, were purchased from AddGene (no. 12259 and no. 12260, respectively).

### Antibodies and reagents.

Mouse monoclonal anti‐pp65 (anti-UL83) (no. ab6503, clone 3A12) was obtained from Abcam, and mouse monoclonal anti‐ICP36 (anti-UL44) (no. MBS530793, clone M612460) was purchased from MyBioSource. Mouse monoclonal anti-hZAP (ZC3HAV1) (no. 66413-1-Ig, clone 1G10B9) and rabbit polyclonal anti-hZAP (no. 16820-1-AP) were obtained from ProteinTech. Mouse monoclonal anti‐actin (A5441, clone AC‐15) was obtained from Sigma‐Aldrich. Rabbit monoclonal anti-myc (no. 2278, clone 71D10) was obtained from Cell Signaling. Mouse monoclonal anti-IE1 (clone 63-27, originally described in reference 71) was a kind gift from Jens von Einem (Institute of Virology, Ulm University Medical Center, Ulm, Germany). Alexa Fluor‐conjugated secondary antibodies were purchased from Invitrogen. The transfection reagent Lipofectamine 2000 was purchased from Life Technologies. Polybrene was obtained from Santa Cruz Biotechnology. Opti-MEM was purchased from Thermo Fisher Scientific. Protease inhibitors (no. 4693116001) were purchased from Roche. Recombinant human IFN-β was purchased from PeproTech (no. 300-02BC).

### Generation of ZAP knockout cells using CRISPR/Cas9-mediated genome editing.

Custom gRNAs targeting the first exon of the ZAP coding region, thus disrupting expression of ZAP-S and ZAP-L, were designed using CRISPOR software (http://crispor.tefor.net) (72) and cloned into the lentiviral pLKO5 vector (kindly provided by Dirk Heckl, Martin Luther University, Halle, Germany). The pLKO5 vector constitutively expresses the introduced gRNA under the control of a U6 promoter. SpCas9 with a P2A cleavage site followed by RFP is under the control of the EF1α short promoter and results in the constitutive expression of SpCas9 and an RFP reporter for cell sorting. Three different gRNAs targeting the ZAP coding region and a nontargeting control gRNA were generated and cloned into the pLKO5 vector via the BsmBI restriction site. ZAP gRNA 1 targets exon 1 at nucleotide 149, ZAP gRNA2 at nucleotide 53, and ZAP gRNA3 at nucleotide 191. The gRNA sequences are as follows: ZAP-g1_FOR, 5′-CACCGGCCGGGCCCGACCGCTTTG; ZAP-g1_REV, 5′-AAACCAAAGCGGTCGGGCCCGGCC; ZAP-g2_FOR, 5′-CACCGCAAAATCCTGTGCGCCCACG; ZAP-g2_REV, 5′-AAACCGTGGGCGCACAGGATTTTGC; ZAP-g3_FOR, 5′-CACCGGCCGGGATCACCCGATCGG; ZAP-g3_REV, 5′-AAACCCGATCGGGTGATCCCGGCC; control-gRNA_FOR, 5′-CACCGGATTCTAAAACGGATTACC; control-gRNA_REV, 5′-AAACGGTAATCCGTTTTAGAATCC. For lentivirus production, HEK 293T cells (730,000 cells per well, 6-well format) were transfected with 400 ng pMD2.G, 1,600 ng psPAX2, and 2,000 ng pLKO5 plasmid (containing the respective gRNA) complexed with Lipofectamine. Then, 16 h posttransfection, medium was changed to lentivirus harvest medium (DMEM h.gl. supplemented with 20% FCS, 1% P/S, and 10 mM HEPES); 48 h posttransfection, lentivirus was harvested and diluted 1:2 with HFF-1 medium, and Polybrene was added to a final concentration of 4 μg/ml. HFF-1 cells were seeded the day before transduction in a 6-well format with 250,000 cells/well. For transduction, HFF-1 medium was replaced by medium containing lentivirus, and cells were transduced by centrifugal enhancement at 684 × *g* and 30°C for 90 min. Next, 3 h postransduction, medium was replenished with fresh HFF-1 medium. Successfully transduced cells were sorted by flow cytometry for RFP signal 72 h postransduction to obtain a cell population devoid of ZAP expression. Cas9-mediated knockout of ZAP was verified by immunoblotting.

### Reconstitution assays.

For reconstitution of ZAP-S or ZAP-L expression in ZAP KO cell lines, lentiviral transduction was performed as described above. Briefly, 2,000 ng pTRIP-IRES-RFP empty vector or pTRIP-IRES-RFP containing codon-optimized C-terminally myc-tagged ZAP-S, ZAP-L, or untagged ZAP-L, together with 400 ng pCMV-VSV-G and 1,600 ng pCMV-gag/pol complexed with Lipofectamine was transfected into HEK 293T cells, and medium was changed to lentivirus harvest medium the next day. Then, 48 h posttransfection, the WT or the indicated ZAP KO HFF-1 cells were lentivirally transduced; 72 h postransduction, cells were counted, and 100,000 cells per well were seeded in a 24-well format. The next day, cells were infected with HCMV WT at a multiplicity of infection (MOI) of 0.1, and infection was enhanced by centrifugation at 684 × *g* for 45 min at 30°C. After centrifugation, cells were incubated at 37°C for 30 min followed by replacement of virus-containing medium with fresh HFF-1 medium. At the indicated time points postinfection, cells were lysed for analysis by immunoblotting or qRT-PCR as described below.

### Immunoblotting.

For the analysis of viral protein kinetics upon HCMV infection, HFF-1 WT or ZAP KO cells (100,000 cells/well in a 24-well format) were infected with HCMV WT at an MOI of 0.1, and the infection was enhanced by centrifugation at 684 × *g* at 30°C for 45 min. The moment when the virus was added to the cells was defined as time point 0. After centrifugation, cells were incubated at 37°C for 30 min followed by replacement of virus-containing medium with fresh HFF-1 medium. Cells were lysed at the indicated time points using radioimmunoprecipitation (RIPA) buffer (20 mM Tris-HCl pH 7.5, 1 mM EDTA, 100 mM NaCl, 1% Triton X‐100, 0.5% sodium deoxycholate, 0.1% SDS). Protease inhibitors were added freshly to all lysis buffers prior to use. Cell lysates and samples were separated by SDS-PAGE and transferred to polyvinylidene difluoride (PVDF) membrane (GE Healthcare) using wet transfer and Towbin blotting buffer (25 mM Tris, 192 mM glycine, 20% [vol/vol] methanol). Membranes were probed with the indicated primary antibodies and respective secondary horseradish peroxidase (HRP)‐coupled antibodies diluted in 5% wt/vol nonfat dry milk or 5% bovine serum albumin (BSA) in Tris-buffered saline with Tween 20 (TBS‐T). Immunoblots were developed using SuperSignal West Pico (Thermo Fisher Scientific) chemiluminescence substrates. Membranes were imaged with a ChemoStar ECL imager (INTAS) and quantified using LabImage 1D software (INTAS).

### Immunofluorescence.

ZAP KO HFF-1 cells were lentivirally transduced as described above. Briefly, 2,000 ng pTRIP-IRES-RFP containing codon-optimized C-terminally myc-tagged ZAP-S or ZAP-L together with 400 ng pCMV-VSV-G and 1,600 ng pCMV-gag/pol complexed with Lipofectamine were transfected into HEK 293T cells, and medium was changed to lentivirus harvest medium the next day. Then, 48 h posttransfection, ZAP KO HFF-1 cells were lentivirally transduced; 72 h postransduction, cells were counted, and 20,000 cells per well were seeded in a μ-Slide 8-well chamber slide (ibidi; no. 80826). The next day, cells were mock-infected or infected with HCMV WT at an MOI of 0.1, and infection was enhanced by centrifugation at 684 × *g* for 45 min at 30°C. After centrifugation, cells were incubated at 37°C for 30 min, followed by replacement of virus-containing medium with fresh HFF-1 medium. Cells were fixed 24 hpi using 4% paraformaldehyde (PFA) in phosphate-buffered saline (PBS) for 20 min at room temperature. Cells were washed three times with PBS, followed by permeabilization using 0.4% Triton X-100 in PBS for 10 min at room temperature. Cells were washed three times with PBS and blocked with 4% BSA in PBS for 45 min. Cells were stained with the indicated primary antibodies and respective secondary antibodies coupled to Alexa488, or Alexa647, and Hoechst (Thermo Fisher Scientific; no. 33342) diluted in 4% BSA in PBS for 45 min at room temperature in the dark. Imaging was done on a Nikon ECLIPSE Ti‐E‐inverted microscope equipped with a spinning disk device (Perkin Elmer Ultraview), and images were processed using Volocity software version 6.2.1 (Perkin Elmer).

### Quantitative RT-PCR.

HFF-1 WT or ZAP KO cells were infected with HCMV WT as described above. Briefly, an MOI of 0.1 was used, and the infection was enhanced by centrifugation at 684 × *g* at 30°C for 45 min, followed by 30 min incubation at 37°C and replacement of virus-containing medium with fresh HFF-1 medium. Cells were lysed in RLT buffer, and RNA was purified using the Analytik Jena RNA isolation kit (845-KS-2040250) following the manufacturer’s protocol. After RNA extraction, 1,500 ng of RNA per sample was used for further processing. DNase treatment and cDNA synthesis were performed with the iScript gDNA Clear cDNA synthesis kit (no. 1725035) following the manufacture’s protocol. Generated cDNA was diluted 1:5 before performing qPCR to obtain 100 μl of cDNA. For quantification of gene transcripts, 5 μl of cDNA per sample was used, and qRT‐PCR was performed using the GoTaq qPCR master mix (Promega, A6001) on a LightCycler 96 instrument (Roche). GAPDH was used for normalization. The following oligo sequences were used: GAPDH_FOR, 5′-GAAGGTGAAGGTCGGAGTC; GAPDH_REV, 5′-GAAGATGGTGATGGGATTTC; UL44_FOR, 5′-CGCGACGTTACTTTGATTTGAG; UL44_REV, 5′-ATTCGGACGCCGACATTAG; UL83_FOR, 5′-AACCAAGATGCAGGTGATAGG; UL83_REV, 5′-AGCGTGACGTGCATAAAGA; TNFRSF10D_FOR, 5′-CTGCTGGTTCCAGTGAATGACG; TNFRSF10D_REV, 5′-TTTTCGGAGCCCACCAGTTGGT; ZMAT3_FOR, 5′-GCTCTGTGATGCCTCCTTCAGT; ZMAT3_REV, 5′-TTGACCCAGCTCTGAGGATTCC.

### Determination of HCMV genome copy numbers.

HFF-1 WT, control, or ZAP KO cells were infected with HCMV WT at an MOI of 0.1 for 2 h at 37°C. After this time, cells were washed once with fresh medium and incubated at 37°C. At the indicated time points, cells were scraped into the supernatants, and cells and supernatant were harvested together. DNA from 200 μl of the samples was extracted using the Qiagen DNeasy blood and tissue kit (no. 69504) following the manufacturer’s protocol. Extracted DNA was diluted 1:10 prior to qPCR for the analysis of HCMV genome copy numbers. HCMV DNA copy numbers were quantified with a real-time quantitative PCR as described previously (73). Copy numbers were harmonized to the 1st WHO International Standard for Human Cytomegalovirus for Nucleic Acid Amplification Techniques (NIBSC, no. 09/162).

### Total proteome analyses using LC-MS/MS.

HFF-1 WT or ZAP KO (250,000 cells/well in a 6-well format) were mock-treated or infected with HCMV WT at an MOI of 0.1, and the infection was enhanced by centrifugation at 684 × *g* at 30°C for 45 min. The moment when the virus was added to the cells was defined as time point 0. After centrifugation, cells were incubated at 37°C for 30 min followed by removal of the virus-containing medium, one wash with DMEM, and replacement with fresh medium. At the indicated time points, cells were washed with PBS once and then collected in 300 μl of fresh PBS per well. Cell pellets were frozen at −70°C. Two wells were combined to obtain a total of 500,000 cells per condition. Quadruplicates of HCMV-infected HFF-1 cells were analyzed at 48 and 72 h postinfection. For each replicate, cells were washed with PBS, lysed in SDS lysis buffer (4% SDS, 10 mM dithiothreitol [DTT], 50 mM Tris/HCl pH 7.6), boiled at 95°C for 5 min, and sonicated (4°C, 10 min, 30 sec on, 30 sec off; Bioruptor). Protein concentrations of cleared lysates were normalized, and cysteines were alkylated with 5.5 mM indole-3-acetic acid (IAA) (20 min, 25°C, in the dark). SDS was removed by protein precipitation with 80% (vol/vol) acetone (−20°C, overnight), and protein pellets were washed with 80% (vol/vol) acetone and resuspended in 40 μl U/T buffer (6 M urea, 2 M thiourea in 10 mM HEPES, pH 8.0). Protein digestion was performed by subsequent addition of 1 μg LysC (3 h, 25°C) and 1 μg trypsin in 160 μl digestion buffer (50 mM ammonium bicarbonate, pH 8.0) at 25°C overnight. Peptides were desalted and concentrated using C_18_ stage tips as described previously (74). Purified peptides were loaded onto a 50-cm reverse-phase analytical column (75 μm diameter; ReproSil-Pur C18-AQ 1.9 μm resin; Dr. Maisch) and separated using an EASY-nLC 1200 system (Thermo Fisher Scientific). A binary buffer system consisting of buffer A (0.1% formic acid in H_2_O) and buffer B (80% acetonitrile, 0.1% formic acid in H_2_O) with a 120-min gradient (5 to 30% buffer B [95 min], 30 to 95% buffer B [10 min], wash out at 95% buffer B [5 min], decreased to 5% buffer B [5 min], and 5% buffer B [5 min]) was used at a flow rate of 300 nl per min. Eluting peptides were directly analyzed on a Q-Exactive HF mass spectrometer (Thermo Fisher Scientific). Data-dependent acquisition included repeating cycles of one MS1 full scan (300 to 1,650 *m/z*, *R* = 60,000 at 200 *m/z*) at an ion target of 3 × 10^6^, followed by 15 MS2 scans of the highest abundant isolated and higher-energy collisional dissociation (HCD) fragmented peptide precursors (*R* = 15,000 at 200 *m/z*). For MS2 scans, collection of isolated peptide precursors was limited by an ion target of 1 × 10^5^ and a maximum injection time of 25 ms. Isolation and fragmentation of the same peptide precursor were eliminated by dynamic exclusion for 20 s. The isolation window of the quadrupole was set to 1.4 *m/z*, and HCD was set to a normalized collision energy of 27%. Raw files were processed with MaxQuant (version 1.6.14.0) using the standard settings and label-free quantification (LFQ) and match between runs options enabled. Spectra were searched against forward and reverse sequences of the reviewed human proteome, including isoforms (UniProtKB, release 01.2019) and of the HCMV proteins (accession number GenBank accession no. EF999921.1) by the built-in Andromeda search engine (75).

The output of MaxQuant was analyzed with Perseus (version 1.6.14.0) (76), R (version 3.6.0), RStudio (version 1.2.1335), and GraphPad Prism (version 7.04). Detected protein groups identified as known contaminants, reverse sequence matches, only identified by site, or quantified in less than 3 out of 4 replicates in at least one condition were excluded. Following log_2_ transformation, missing values were input for each replicate individually by sampling values from a normal distribution calculated from the original data distribution (width = 0.3 · S.D., downshift = −1.8 · S.D.). Differentially expressed protein groups between biological conditions were identified via two-sided Student’s *t* tests corrected for multiple hypotheses testing applying a permutation-based false-discovery rate (FDR) (250 randomizations).

### Total transcriptome analyses (RNA sequencing).

HFF-1 WT, control, and two independent ZAP KO cells (250,000 cells/well in a 6-well-plate format) were untreated, mock-treated, or infected by centrifugal enhancement at 684 × *g* and 30°C for 45 min with HCMV WT at an MOI of 0.1. The moment when the virus was added to the cells was defined as time point 0. After centrifugation, cells were incubated at 37°C for 30 min followed by removal of the virus-containing medium and washed with fresh DMEM once. Medium was then replaced with previously conditioned medium (medium in which the cells were originally seeded was kept at 37°C during the infection time). Cells were lysed at the indicated time points using TRIzol for 2 min at room temperature and kept at −70°C. Two wells were combined to obtain around 500,000 cells per sample. Total RNA was isolated using the RNA clean and concentrator kit (Zymo Research) according to the manufacturer’s instructions. Sequencing libraries were prepared using the NEBNext Ultra II directional RNA library prep kit for Illumina (NEB; catalog no. E7760) following polyA RNA enrichment (NEB; catalog no. E7490) with 9 cycles of PCR amplification and sequenced on a HiSeq 4000 1 × 50-cycle flow cell.

Alignments were done using HISAT 2 (77). Sequencing reads were aligned to the hg19 version of the human genome using standard parameters, using the Refseq gtf file downloaded from the UCSC genome browser. Reads were then quantified using QuasR (78) and the above-mentioned gtf file or the HCMV TB40/E annotation (GenBank accession number MF871618). Differential expression and corresponding *P* values were calculated using edgeR (79). Plots were created using ggplot2 (80) and pheatmap (version 1.0.12) (https://cran.r-project.org/web/packages/pheatmap/index.html).

### SLAM sequencing.

HFF-1 WT or ZAP KO (g3) cells (250,000 cells/well in a 6-well format) were left untreated or infected with HCMV WT at an MOI of 0.1, and the infection was enhanced by centrifugation at 684 × *g* at 30°C for 45 min. The moment when the virus was added to the cells was defined as time point 0. After centrifugation, cells were incubated at 37°C for 30 min followed by removal of the virus-containing medium, one wash with DMEM, and replacement with fresh medium. Newly synthesized RNA was labeled with 4-thiouridine (4sU) for 1 h prior to cell lysis. Cells were lysed at the indicated time points using TRIzol for 5 min at room temperature and kept at −70°C. Two wells were combined to obtain around 500,000 cells per sample. Total RNA was isolated using the DirectZOL kit (Zymo Research) according to the manufacturer’s instructions, including the optional on-column DNase digestion. The 4sU alkylation reaction was essentially performed as published before (39). Briefly, 7.5 to 15 μg total RNA was incubated in 1× PBS (pH 8) containing 50% dimethyl sulfoxide (DMSO) and 10 mM IAA at 50°C for 15 min. The reaction was quenched with 100 mM DTT, and RNA was purified using the RNeasy kit (Qiagen). The quality and integrity of the total RNA were controlled on a 5200 fragment analyzer system. The RNA sequencing library was generated from 100 ng total RNA using a NEBNext single cell/low input RNA library according to the manufacture’s protocols. The libraries were sequenced on an Illumina NovaSeq 6000 device using a NovaSeq 6000 S1 reagent kit (300 cycles, paired-end run, 2 × 150 bp) with an average of 40 × 10^6^ reads per RNA sample.

SLAM-seq was performed in duplicates to identify newly synthesized and total RNA using GRAND-SLAM. Sequencing adapters (AGATCGGAAGAGCACACGTCTGAACTCCAGTCA, AGATCGGAAGAGCGTCGTGTAGGGAAAGAGTGT) were trimmed using Trimmomatic (version 0.39) (81). Reads were mapped to a combined index of the human genome (Hg38/Ensembl version 90) and the HCMV genome (GenBank accession number EF999921.1) using STAR (version 2.5.3a) with the parameters –outFilterMismatchNmax 20 –outFilterScoreMinOverLread 0.3 –outFilterMatchNminOverLread 0.3 –alignEndsType Extend5pOfReads12 –outSAMattributes nM MD NH. We used GRAND-SLAM (version 2.0.5d) (40) to estimate the new-to-total RNA ratios. We only used the parts of the reads that were sequenced by both mates in a read pair (parameter -double) for the estimation. New RNA was computed by multiplying total RNA with the maximum *a posteriori* estimate of the new-to-total RNA ratio. For further analyses, we removed all cellular genes that had less than 1 transcript per million transcripts (TPM) in more than 6 (cellular genes) or 2 (viral genes) samples. To remove artifacts due to imprecise quantification, we furthermore removed all viral genes with less than 100 new reads. Log_2_ fold changes were estimated using PsiLFC (82) with uninformative prior (corresponding to no pseudocounts). Normalization factors were computed from total RNA such that the median log_2_ fold change was 0 and applied to both total and new RNA.

### Enhanced cross-linking and immunoprecipitation (eCLIP) sequencing.

HFF-1 WT cells (50 × 10^6^ cells) were infected with HCMV WT at an MOI of 1 for 3 h at 37°C and 7.5% CO_2_. After this time, cells were washed once with fresh DMEM and replace with fresh medium. At 20 hpi, the cells were UV cross-linked using a BLX-254 VILBER cross-linker equipped with 254-nm light bulbs. Briefly, the culture medium was removed, and cells were washed once with ice-cold PBS followed by cross-linking on ice. Cross-linking was performed at 254 nm and 0.8 J/cm^2^ UV light. Cells were collected by scraping using ice-cold PBS, followed by centrifugation at 400 × *g* at 4°C for 5 min. The cell pellet was washed once with ice-cold PBS and frozen down in liquid nitrogen. Frozen cell pellets were lysed in 50 mM Tris-HCl pH 7.4, 150 mM NaCl, 1 mM EDTA, 1% (vol/vol) NP-40, 0.5% NaDeoxycholate, 0.25 mM TCEP [Tris(2-carboxyethyl)phosphine hydrochloride], and complete EDTA-free protease inhibitor cocktail. Subsequent steps were performed as described in the eCLIP protocol (42), with the following modifications. Immunoprecipitates were washed two times in 1 ml CLIP lysis buffer and two times in IP wash buffer (50 mM Tris-HCl pH 7.4, 300 mM NaCl, 1 mM EDTA, 1% [vol/vol] NP-40, 0.5% sodium deoxycholate, 0.25 mM TCEP), followed by two washes in 50 mM Tris-HCl pH 7.4, 1 mM EDTA, and 0.5% (vol/vol) NP-40. All other steps were carried out as described in the eCLIP method. Briefly, following cell lysis, unprotected RNA was digested, and ZAP was immunoprecipitated using a specific antibody. Cross-linked protein-RNA complexes were separated by SDS-PAGE, followed by nitrocellulose transfer. Bands migrating at the expected size range were excised, and recovered RNA fragments were converted into a cDNA library according to the eCLIP procedure (42). Sequencing was performed using the NextSeq 500 technology. For immunoprecipitation reactions, a specific antibody against ZAP (ZC3HAV1) (Proteintech, 16820-1-AP) was used.

Paired-end sequencing reads from eCLIP experiments were trimmed using a custom Python script that simultaneously identified the umi-molecular identifier (UMI) associated with each read. Trimmed reads were then aligned to the HCMV genome (GenBank accession number EF999921.1) and the human genome (Hg38/Ensembl v100) using the Burrows-Wheeler Aligner (83) (version 0.7.15-r1140). Next, we removed PCR duplicates using the UMI-aware deduplication functionality in Picard’s MarkDuplicates, and the generated .bam files were then split by strand using “samtools view -f/-F 16” and converted to .bw (bigwig format) using bamCoverage (84). Finally, ZAP binding sites were identified as peaks that were enriched relative to a size-matched input control. MACS2 (85) (version 2.2.7.1) callpeak was used with the parameters -f BAM –keep-dup all –nomodel –extsize 50 –d-min 5 –scale-to small -B.

For comparing the binding site with gene expression (Fig. 6D and Fig. S6), all binding sites overlapping the annotated region of the gene were identified using custom scripts, and the binding site with the strongest enrichment was used. For the binding preference analysis, the set of strongly expressed genes defined by our SLAM-seq data was used (see SLAM sequencing section). For each of these genes, the sequences of the transcript isoform that had the most peaks (and the longest in case more than one transcript had the same number of peaks) were extracted. Within each of these transcripts, dinucleotide counts inside and outside the whole binding site regions defined by MACS2 were identified with a custom script for Fig. 6B. Furthermore, the position directly upstream of the 5′ end of the second read of an eCLIP read pair was considered to be the site of cross-linking. Sequences ±100 nucleotides (nt) of the main cross-linking site (defined as the position in a transcript isoform having the maximal number of cross-linking events) were extracted from the transcript sequences (excluding introns), and dinucleotides were counted for each position using custom scripts for Fig. 6C. DREME (version 5.0.1) (43) was used to identify sequence motifs (with the parameter -norc) in these sequences.

### Statistical analyses.

For qRT-PCR and genome copy number quantification, differences between data sets were evaluated after log transformation with Student’s *t*‐test (unpaired, two‐tailed), using GraphPad Prism version 5.0 (GraphPad Software, San Diego, CA). *P* values of < 0.05 were considered statistically significant.

### Data availability.

The mass spectrometry proteomics data have been deposited in the ProteomeXchange Consortium via the PRIDE (86) partner repository with the data set identifier PXD023559.

The raw sequencing data, including SLAM-seq, RNA-seq, and CLIP-seq, have been deposited in the NCBI GEO database, with the accession number GSE159853.
