# Supplementary figures and images for: The Zinc Finger Antiviral Protein ZAP Restricts Human Cytomegalovirus and Selectively Binds and Destabilizes Viral UL4/UL5 Transcripts
Source: mBio. 2021 May 4;12(3):e02683-20. doi: 10.1128/mBio.02683-20 (PMC8263000; doi:10.1128/mBio.02683-20)

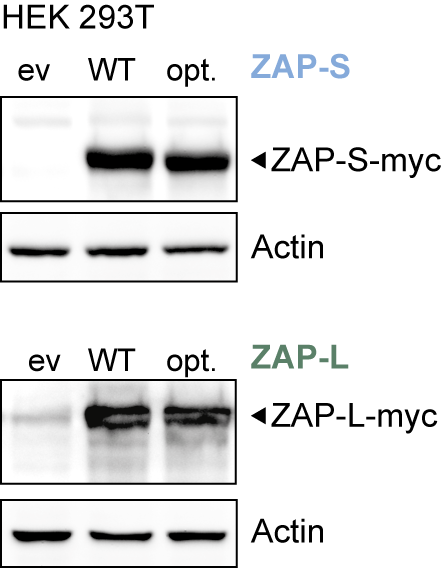

Supplement: FIG S1 [file mbio.02683-20-sf001.tif]

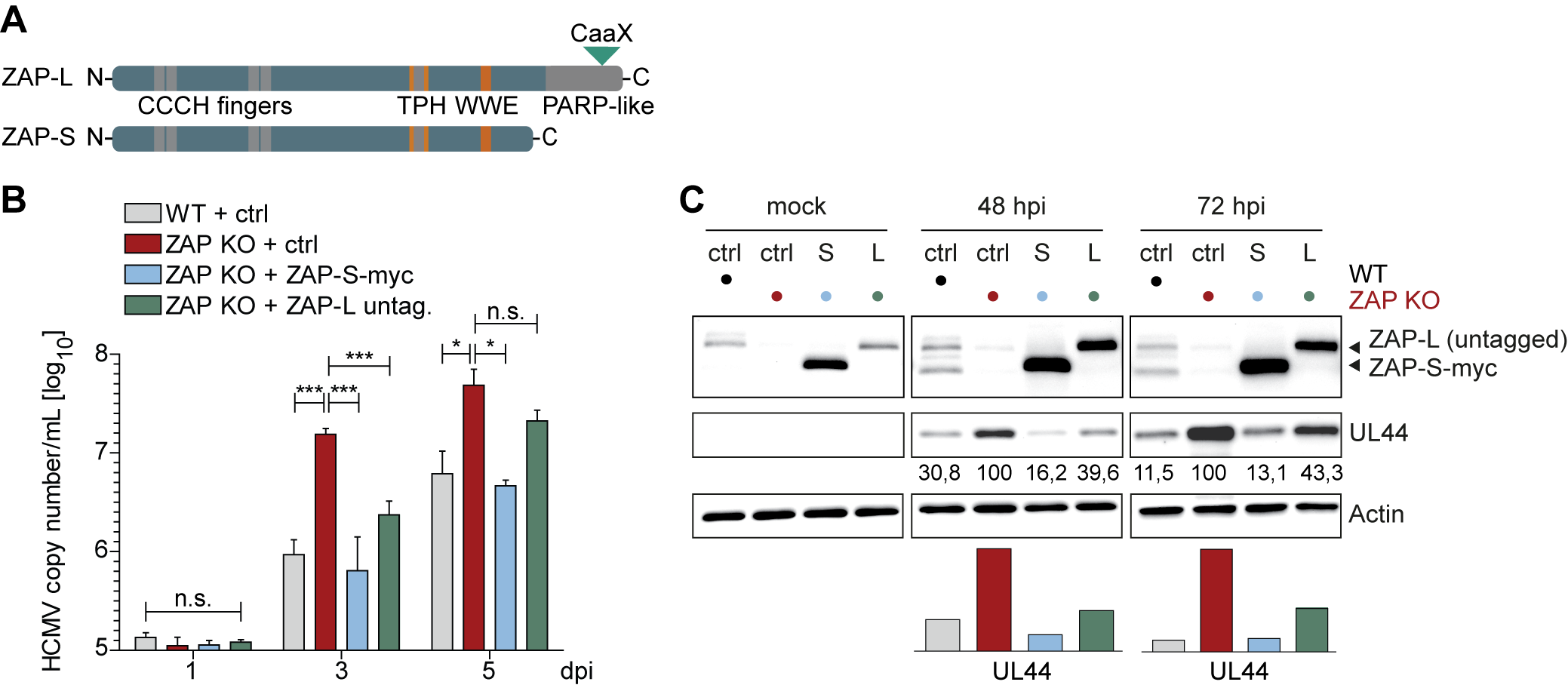

Supplement: FIG S2 [file mbio.02683-20-sf002.tif]

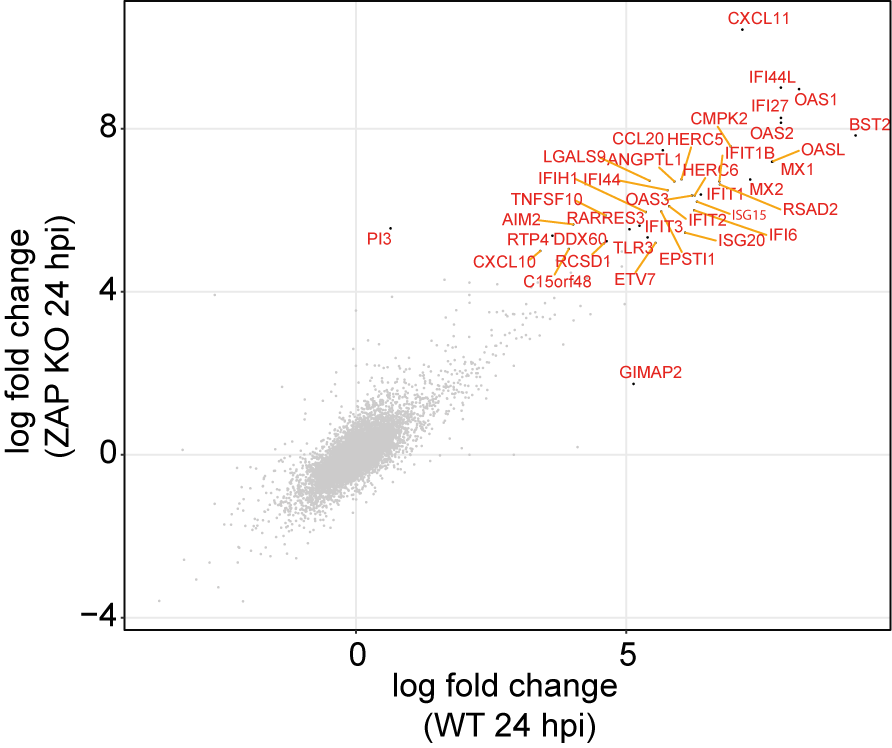

Supplement: FIG S3 [file mbio.02683-20-sf003.tif]

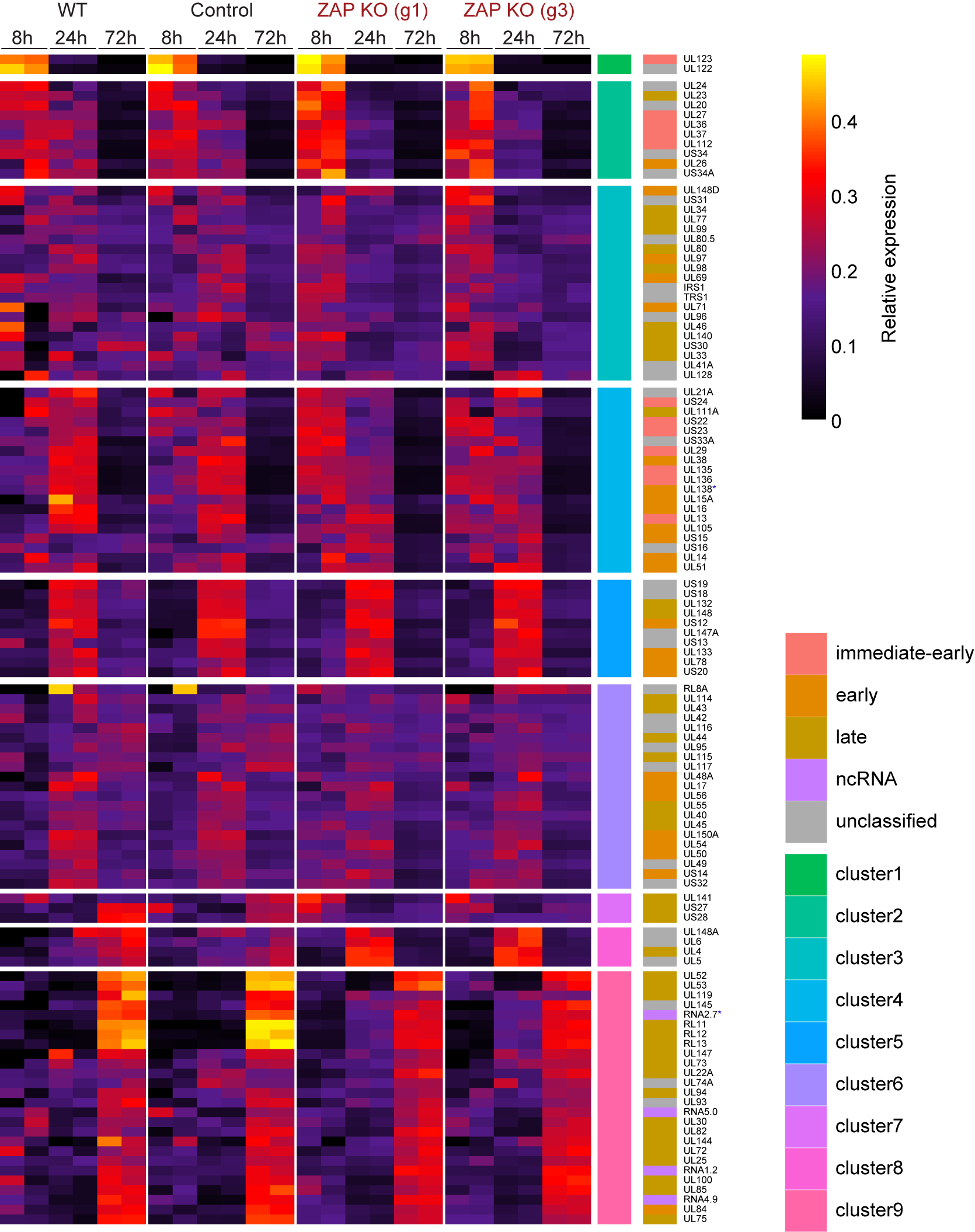

Supplement: FIG S4 [file mbio.02683-20-sf004.tif]

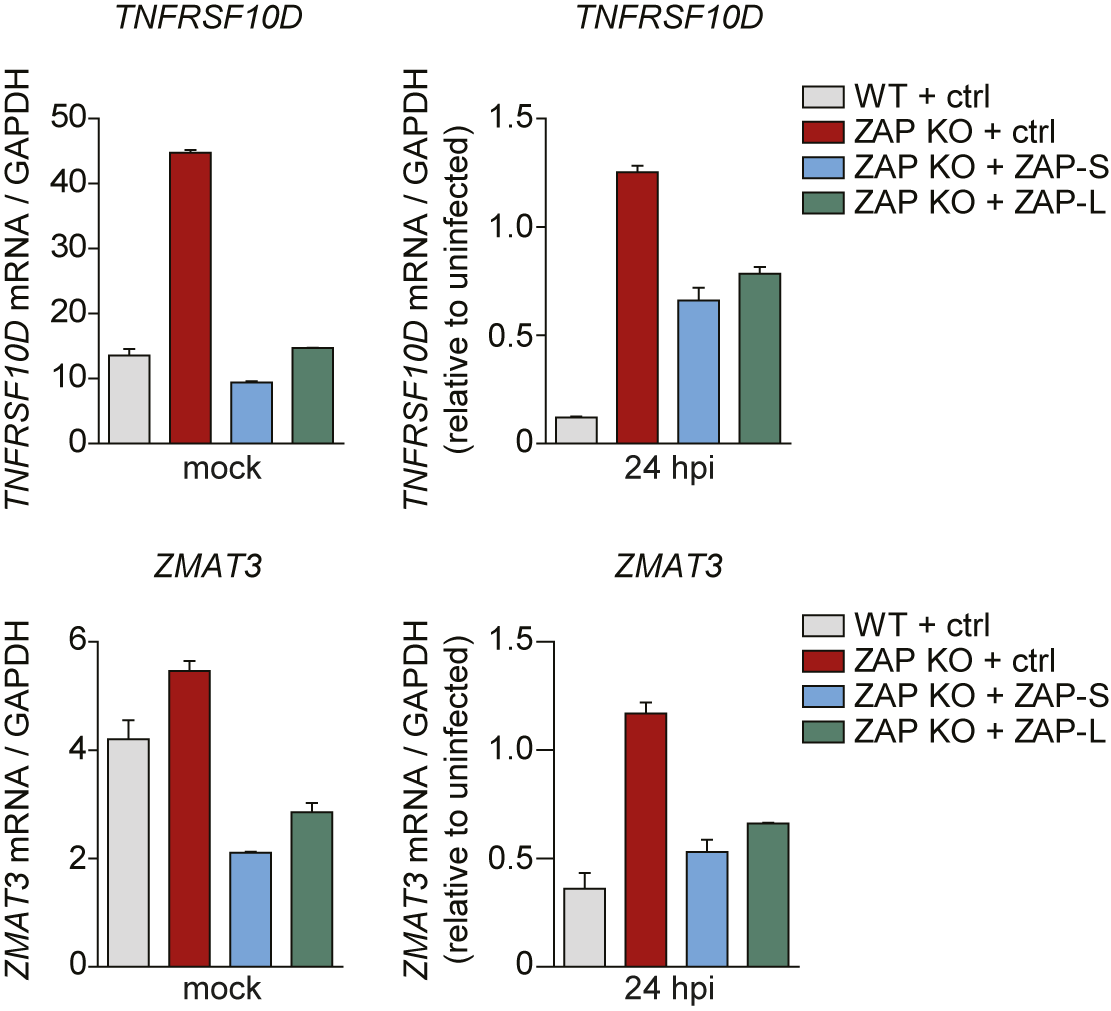

Supplement: FIG S5 [file mbio.02683-20-sf005.tif]

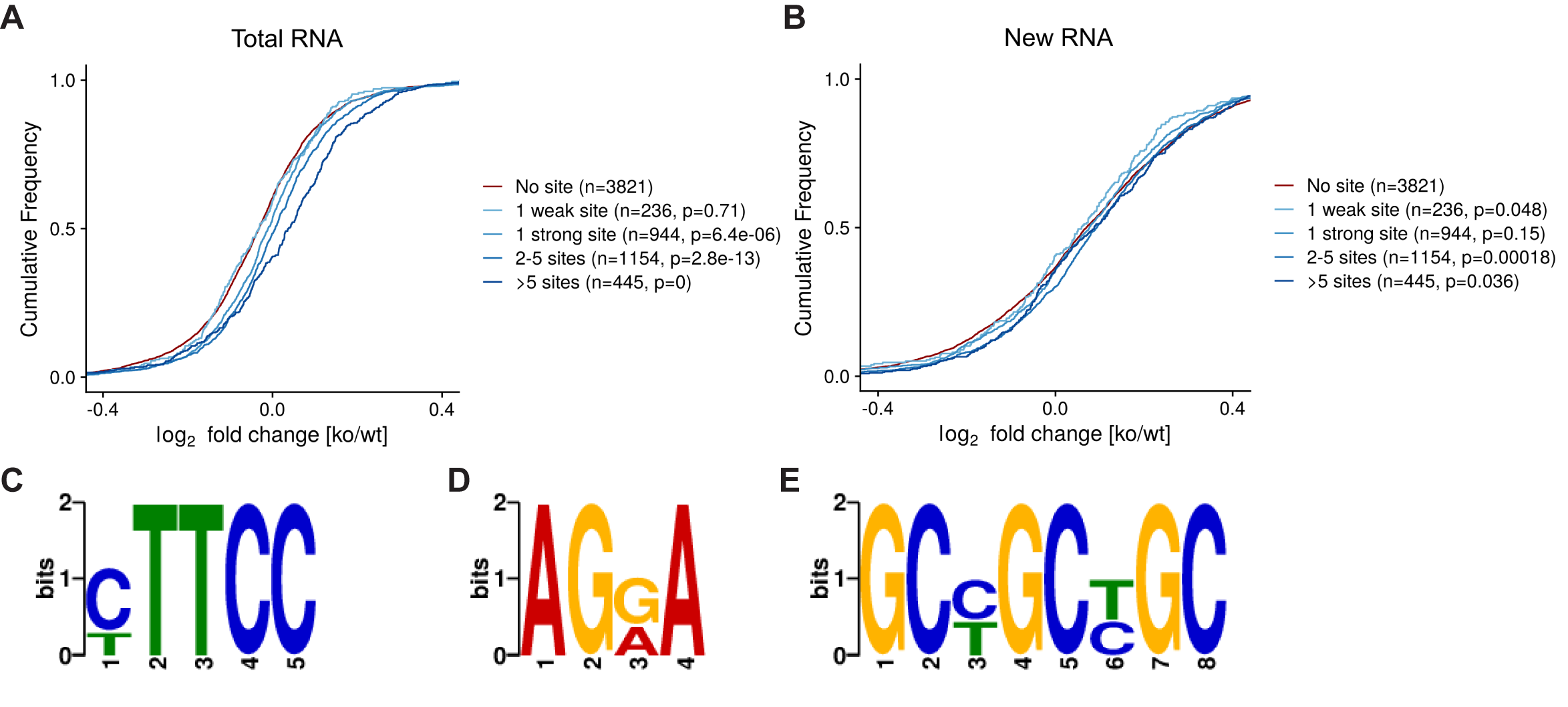

Supplement: FIG S6 [file mbio.02683-20-sf006.tif]
